# A cyclic peptide-grafted Fc with hepatocyte growth factor functionality ameliorates hepatic fibrosis in a non-alcoholic steatohepatitis mouse model

**DOI:** 10.1016/j.isci.2024.110426

**Published:** 2024-07-02

**Authors:** Nichole Marcela Rojas-Chaverra, Ryu Imamura, Hiroki Sato, Toby Passioura, Emiko Mihara, Tatsunori Nishimura, Junichi Takagi, Hiroaki Suga, Kunio Matsumoto, Katsuya Sakai

**Affiliations:** 1 Division of Tumor Dynamics and Regulation, Cancer Research Institute, Kanazawa University, Kanazawa 920-1192, Japan; 2 WPI-Nano Life Science Institute (WPI-NanoLSI), Kanazawa University, Kanazawa 920-1192, Japan; 3 School of Chemistry, School of Life and Environmental Sciences, and Sydney Analytical, The University of Sydney, Sydney NSW 2006, Australia; 4 Department of Chemistry, Graduate School of Science, The University of Tokyo, Tokyo 113-0033, Japan; 5 Laboratory of Protein Synthesis and Expression, Institute for Protein Research, Osaka University, Suita 565-0871, Japan; 6 Division of Cancer Biology, Nagoya University Graduate School of Medicine, Nagoya 466-8550, Japan

**Keywords:** Fibrosis, Peptides, Biomimetics, Model organism

## Abstract

The regenerative functions associated with cytokines and growth factors have immense therapeutic potential; however, their poor pharmacokinetics, resulting from structural features, hinder their effectiveness. In this study, we aimed to enhance the pharmacokinetics of growth factors by designing receptor-binding macrocyclic peptides through *in vitro* mRNA display and grafting them into loops of immunoglobulin’s crystallizable region (Fc). As a model, we developed peptide-grafted Fc proteins with hepatocyte growth factor (HGF) functionality that exhibited a prolonged circulation half-life and could be administered subcutaneously. The Fc-based HGF mimetic alleviated liver fibrosis in a mouse model fed a choline-deficient high-fat diet, which induces hepatic features of non-alcoholic steatohepatitis, including fibrosis, showcasing its potential as a therapeutic intervention. This study provides a basis for developing growth factor and cytokine mimetics with improved pharmacokinetics, expanding their therapeutic applications.

## Highlights

• *In vitro* mRNA display identifies Met receptor-binding macrocyclic peptides
• Bivalent display on Fc converts Met-binding peptides to agonist Fc(mML1)B3
• Fc(mML1)B3 enhances bioavailability over native ligand HGF
• Fc(mML1)B3 treatment alleviates hepatic fibrosis in a mouse NASH model

## Graphical abstract

**Figure:**
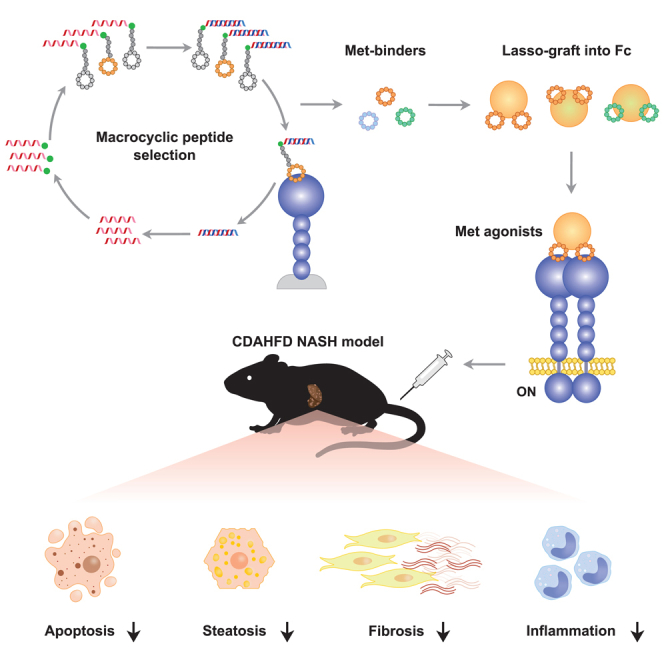

## Introduction

Cytokines and growth factors are used clinically in therapy against cancer, autoimmune diseases, and viral infections.^1^ Ongoing investigations are exploring their potential use in neurogenesis and the repair of brain tissue.^2^ However, the therapeutic utilization of cytokines and growth factors has been hindered by factors such as low stability, pleiotropy, and poor pharmacokinetics.^3^ Researchers are developing surrogate agonists structurally unrelated to the natural ligands to overcome these limitations associated with the inherent structure of cytokines and growth factors. Examples include small antibody domains^4^ and DNA aptamers.^5^

Macrocyclic peptides with molecular masses ranging from 500 to 2,500 Da have emerged as promising molecules due to their antibody-like binding affinity and specificity and the ability to target unique chemical spaces.^6^^,^^7^ The random nonstandard peptide integrated discovery (RaPID) system is a technology that involves the *in vitro* synthesis of over 10^12^ compound libraries of thioether-closed macrocyclic peptides, which are then screened against a protein target of interest.^8^^,^^9^ In a recent study, we achieved the development of designed surrogate growth factor receptor agonists with significantly improved half-lives and the ability to penetrate the blood-brain barrier. This achievement was accomplished by genetically inserting RaPID-identified receptor-binding macrocyclic peptides into the crystallizable region (Fc) of an immunoglobulin or anti-transferrin receptor antibody.^10^ This approach, called lasso-graft (LG), combines the binding function of peptides derived from mRNA display with the host protein function, such as Fc.^10^^,^^11^^,^^12^

Non-alcoholic fatty liver disease (NAFLD) is a chronic condition marked by hepatic lipid accumulation, including non-alcoholic fatty liver (NAFL) and non-alcoholic steatohepatitis (NASH). NASH involves fat accumulation, hepatocellular damage, inflammation, and progressive fibrosis, where extracellular matrix proteins replace the damaged normal tissue.^13^^,^^14^ If untreated, NASH can progress to severe complications such as hepatocellular carcinoma and cirrhosis, leading to an increased mortality rate.^14^ Excessive lipid accumulation in the liver triggers inflammatory processes that can have detrimental effects on liver health. These effects include the dysfunction of cellular organelles, the activation of hepatic stellate cells, progressive fibrosis, and cell death.^15^ The prevalence of NAFL is approximately 25%, with a higher occurrence among older patients.^14^^,^^16^ NASH is estimated to affect 41.6%–68.3% of individuals diagnosed with NAFLD^14^ and is expected to increase in prevalence as obesity and metabolic syndrome also rise.^17^ Despite these concerning statistics, no drugs are approved by the US Food and Drug Administration (FDA) or European Medicines Agency (EMA) for NASH.^18^

Hepatocyte growth factor (HGF) induces the dimerization of the receptor tyrosine kinase Met, resulting in the autophosphorylation of Met tyrosine residues and subsequent activation of downstream effectors. This activation promotes cell growth, survival, and migration.^19^^,^^20^^,^^21^^,^^22^^,^^23^ Met signaling plays a role in suppressing liver steatosis and NASH, as evidenced by studies on hepatocyte-specific Met knockout (KO) mice, which exhibit liver steatosis.^24^ Moreover, when subjected to a NASH-inducing diet, hepatocyte-specific Met KO mice^21^ or Kupffer cells/myeloid cell-specific Met KO mice^23^ develop more severe NASH and liver fibrosis. The therapeutic potential of HGF has been demonstrated in preclinical models of several diseases, including NASH.^21^^,^^23^^,^^25^^,^^26^^,^^27^^,^^28^ The proposed mechanisms of HGF action in NASH encompass multiple pathways, such as promoting lipid mobilization from the liver,^29^ regulating lipogenesis,^30^ inducing fibrinolysis,^31^ triggering the antioxidant response,^32^ and inhibiting inflammation.^33^ However, HGF exhibits a short biological half-life, necessitating repeated intravenous administration at high concentrations to achieve a sustained effect.^25^^,^^26^^,^^27^^,^^29^^,^^33^ Consequently, to overcome the limitations of recombinant HGF and increase its clinical therapeutic potential, alternative Met agonists that require fewer injections and are suitable for subcutaneous administration are needed.

In this study, we developed HGF mimetics by incorporating a Met-binding macrocyclic peptide into the Fc protein using LG. The Fc-dimer structure contained eight LG-compatible loops in a single chain, enabling receptor dimerization and activation. The Fc-based HGF mimetic exhibited advantageous characteristics such as high expression, prolonged half-life in the bloodstream, and subcutaneous administration —attributes inherent to the Fc protein. Moreover, when administered subcutaneously once a week, the Fc-based HGF mimetic demonstrated effectiveness in promoting the reduction of lipid accumulation, fibrosis, and macrophage aggregation in the liver of mice fed a choline-deficient, L-amino acid-defined, high-fat diet (CDAHFD), a diet that induces hepatic features similar to those observed in human NASH.^34^ Thus, the LG present on the immunoglobulin Fc has the potential to serve as a versatile platform for designing growth factor and cytokine mimetics with favorable pharmacokinetic properties. These LG-based mimetics could offer novel treatment options for chronic diseases and central nervous system disorders that are challenging to target by natural ligands.

## Results

### Random non-standard peptide integrated discovery selection of macrocyclic peptides targeting Met receptor

**Figure 1:**
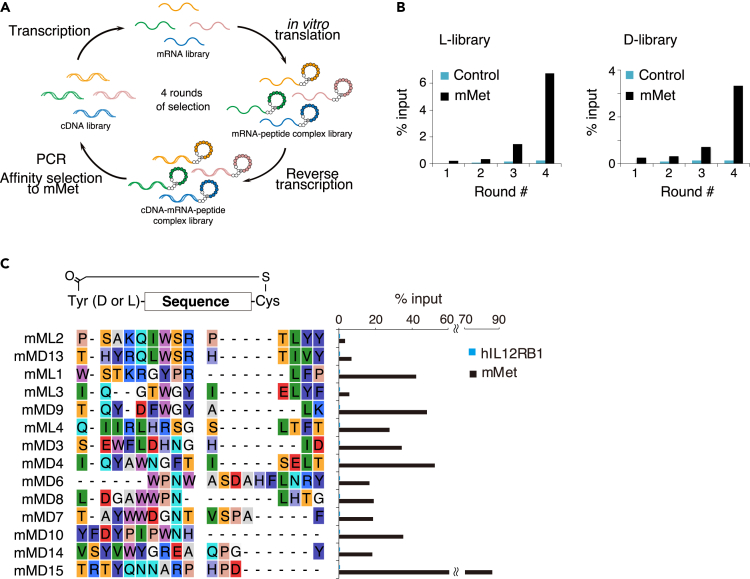
RaPID selection of macrocyclic peptides against mouse Met ectodomain (A) Illustration of RaPID. Mouse Met (mMet)-binding peptides were identified from an mRNA library encoding > 10^12^ macrocyclic peptides by affinity selection for mMet ectodomain-immobilized beads. (B) Enrichment of mMet-binding peptides by selection from libraries initiated with *N*-chloroacetyl-*l*-Tyr (L-library) or *N*-chloroacetyl-*d*-Tyr (D-library). Bars represent the cDNA recovery rates from macrocyclic peptide–mRNA complexes binding to mMet-Fc beads (mMet) or Fc beads (Control). (C) Sequence alignment of mMet-binding peptides identified by RaPID selection (Left). mML, mMet-binding peptides from L-library; mMD, mMet-binding peptides from D-library. Specific binding of mMet-binding peptides to mMet beads but not human interleukin 12 receptor b1-Fc beads (hIL12RB1) (Right graph). Binding of *in vitro* translated macrocyclic peptide–mRNA complexes to mMet-Fc beads or hIL12RB1-Fc beads was detected by PCR. Bars represent the recovery rates.

Using RaPID, we have identified macrocyclic peptides capable of binding to the human Met receptor ectodomain.^10^^,^^35^ However, the high species-specificity of our macrocyclic peptides to human Met did not allow us to evaluate them on mouse disease models. Therefore, we thought to identify macrocyclic peptides that bind to the mouse Met receptor ectodomain (mMet_ECD_) (Figure 1A). We used two peptide libraries initiated with *N*-chloroacetyl-L-Tyr (L-library) or *N*-chloroacetyl-D-Tyr (D-library), which yielded thioether-closed macrocyclic peptides by reacting spontaneously with a cysteine residue downstream of the translated 4–15 random amino acids. The translated macrocyclic peptides, connected with their cognate mRNAs, were affinity-selected against the mMet_ECD_-Fc fusion protein. mMet-binding species were isolated, and their mRNAs/cDNAs were enriched by PCR. Recovery of mMet_ECD_-binding species increased after the 3rd to 4th round of selection in both libraries (Figure 1B). After the 4th round, the enriched cDNA pools of the mMet_ECD_-binding macrocyclic peptides were sequenced, and 14 candidates were chosen based on frequencies and sequence variations (Figure 1C). To evaluate the binding of the 14 macrocyclic peptides to mMet_ECD_, they were subjected to *in vitro* translation and assessed for binding to mMet_ECD_-Fc protein or human interleukin 12 receptor b1 (hIL12RB1)-Fc protein. The 14 macrocyclic peptides bound specifically to mMet_ECD_-Fc and not to hIL12RB1-Fc (Figure 1C).

### Hepatocyte growth factor mimetics construction by lasso-graft of mMet-peptides into Fc loops

**Figure 2:**
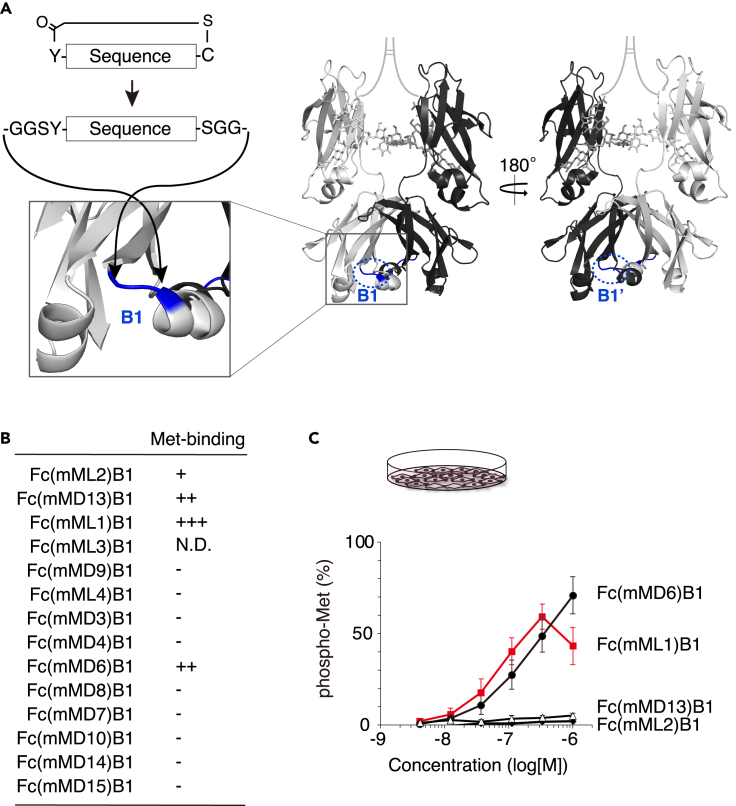
Met agonists were generated by the insertion of mMet binders into Fc loop (A) The pharmacophore sequences of mMet-binding peptides were inserted into B1 and B1′ loops of human IgG1 Fc protein (PDB ID: 1h3w). (B) Binding of peptide-grafted Fc variants with mMet-Fc was evaluated by pull-down assay. N.D., not determined. (C) Cellular Met activation by peptide-grafted Fc variants. AML12 immortalized mouse hepatocytes were treated with Fc variants for 10 min, and Met activation was quantified *in situ* using an anti-phospho-Met (Tyr1234/1235) antibody. The results are shown as the mean ± SD. (*n* = 3 for Fc(mML1)B1, *n* = 6 for others, independent experiments) percentage (%) of phospho-Met relative to maximum Met phosphorylation induced by 1 nM HGF.

To construct HGF mimetics, the pharmacophore sequence of each mMet-binding peptide was inserted into one of the surface-exposed loops of the human IgG1 Fc (B1 loop) (Figure 2A). As Fc is a disulfide-linked dimer, two Met-binding peptides were symmetrically displayed on one Fc molecule, which may induce Met dimerization and activation.^10^^,^^36^ Fc variants with the insertion of a peptide at the B1 loop were named in the format Fc(peptide name)B1. The expression of 13 out of 14 peptide-inserted Fc variants confirmed the feasibility of LG (Figure 2B). The 13 Fc variants were examined for binding with the mMet_ECD_-Fc fusion protein by pull-down assay. Fc(mML1)B1, Fc(mML2)B1, Fc(mMD6)B1, and Fc(mMD13)B1 were found to bind to mMet-Fc (Figure 2B).

Next, we examined whether these Met-binding Fc variants have mMet agonist activity in AML12 immortalized mouse hepatocytes compared to the native ligand, HGF. Cellular Met activation was quantified *in situ* using an anti-phospho-Met (Tyr 1234/1235) antibody. Fc(mML1)B1 exhibited mMet-agonistic activity at lower concentrations than Fc(mMD6)B1 (Figure 2C). Fc(mML1)B1 at 330 nM showed 50.1% of the maximum Met phosphorylation induced by 1 nM HGF (Figure 2C).

**Figure 3:**
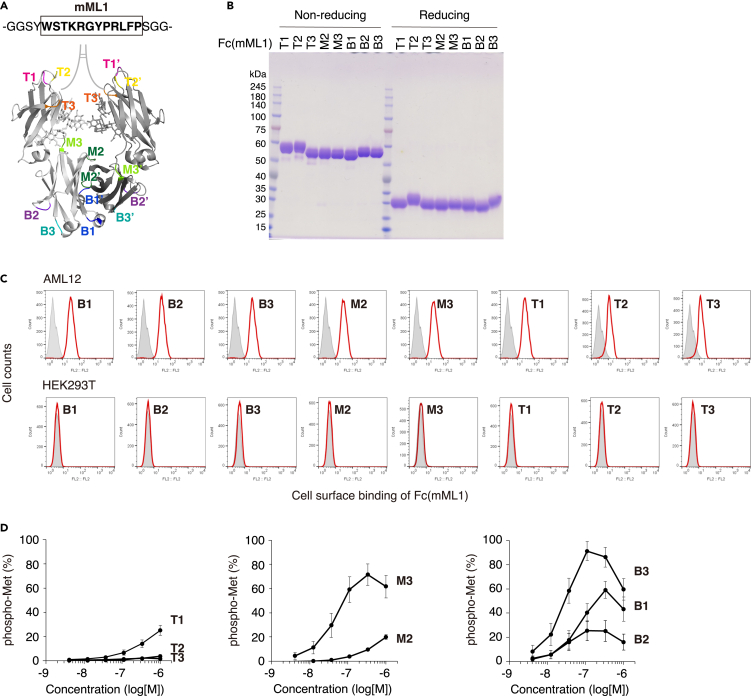
Optimization of graft position of Fc(mML1) for Met agonist activity (A) The mML1 sequence was inserted into each structural loop (T1/T1′∼B3/B3′, colored) of the human IgG1 Fc protein. (B) SDS-PAGE of purified Fc(mML1) variants. (C) Fc(mML1) variants showed similar binding to the Met receptor on AML12 cells. The binding of Fc(mML1) variants to AML12 mouse hepatocytes (above) or HEK293T human cells (below) was detected by flow cytometry. Gray, Fc control; Redline, Fc(mML1) variants. (D) Fc(mML1) variants showed differential Met activation in AML12 cells. Cellular Met activation was quantified *in situ* using an anti-phospho-Met (Tyr1234/1235) antibody. The results are shown as the mean ± SD. (*n* = 3, independent experiments) percentage (%) of phospho-Met relative to maximum Met phosphorylation induced by 1 nM HGF. Fc(mML1)B3 showed the best agonist activity.

To optimize the mML1 sequence graft site for mMet agonist activity, we performed LG of mML1 into the eight loops of Fc (Figure 3A). All Fc(mML1) variants were expressed like control Fc and isolated with >95% purity (Figure 3B). The Fc(mML1) variants were incubated with mouse AML12 cells or human HEK293T cells to examine their binding to the Met receptor on the cell surface and flow cytometry was performed (Figure 3C). The specific binding of Fc(mML1) variants to AML12 cells but not to HEK293T cells suggested that they bound to the mMet receptor. Except for Fc(mML1)T2 and Fc(mML1)T3, which had slightly lower binding capacities (Figure 3C), Fc(mML1) variants showed similar binding to AML12 cells. Despite the similar binding of Fc(mML1) variants to the mMet, their ability to activate the mMet in AML12 cells was highly variable (Figure 3D). B3 was the most suitable site for grafting the mML1 sequence for Met agonist activity (half maximal effective concentration [EC_50_] = 30.2 nM) and showed 91.2% ± 7.9% maximal activation of Met phosphorylation, comparable to induction by HGF. These results suggest that the ability of Fc(mML1) variants to induce proper Met dimer formation, which depends on the grafting position, is the primary determinant of the agonist efficiency.

**Figure 4:**
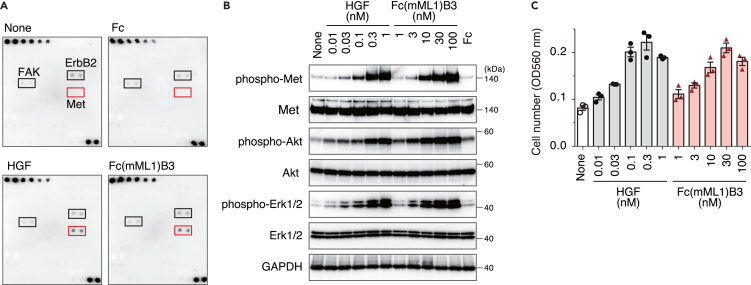
*In vitro* Met activation and cellular responses of Fc(mML1)B3 (A) Phospho-receptor tyrosine kinase arrays demonstrating the selectivity of Fc(mML1)B3 and HGF for Met. AML12 cells were stimulated with HGF (1 nM), Fc(mML1)B3 (50 nM), or Fc (50 nM). (B) Cellular signaling in AML12 cells treated with HGF, Fc(aMD4)B3, or control Fc (100 nM). The lysates were analyzed by Western blotting. (C) Dose-dependent proliferation of AML12 cells induced by HGF or Fc(mML1)B3 determined by MTS assay. The results are shown as the mean ± SD (*n* = 3, replicated wells). See also Figures S1 and S2.

Since the B3 loop was the best grafting position and showed the best Met phosphorylation, we evaluated the Fc(mML1)B3 variant *in vitro* and *in vivo* behavior. *In vitro* experiments, the tyrosine phosphorylation of 71 receptors in AML12 mouse hepatocytes showed that Fc(mML1)B3 and HGF had specificity for the Met receptor (Figure 4A; Figure S1). Next, we compared cell signaling and proliferation of AML12 cells induced by Fc(mML1)B3 or HGF (Figures 4B and 4C). Fc(mML1)B3 induced the phosphorylation of Met and downstream Akt and Erk1/2 at 3–100 nM with activation levels comparable to those caused by HGF (Figures 4B; Figure S2). Furthermore, Fc(mML1)B3 induced AML12 cell proliferation similar to HGF (EC_50_: 13.9 nM, maximum relative to HGF-induced proliferation: 94.6% ± 8.4%) (Figure 4C).

### Pharmacokinetics and Met activation *in vivo* by lasso-graft-based hepatocyte growth factor mimetic

**Figure 5:**
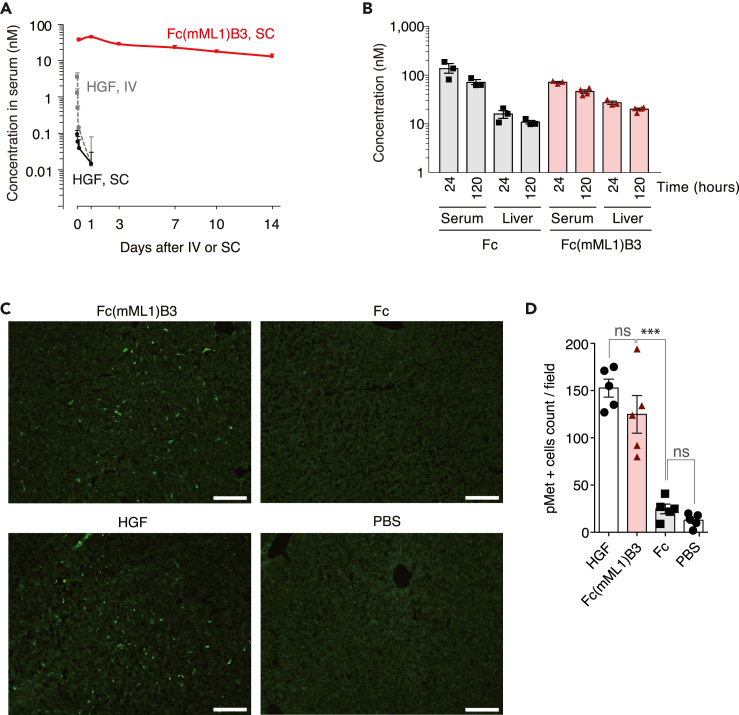
Pharmacokinetics and *in vivo* Met activation of Fc(mML1)B3 (A) Fc(mML1)B3 showed a long biological half-life in the circulation of mice. Serum concentrations of Fc(mML1)B3 or HGF after a single tail vein injection (IV) or subcutaneous (SC) injection of Fc(mML1)B3 (5.0 mg/kg) or HGF (2.0 mg/kg) in wild-type C57BL/6 mice. The results are shown as the mean ± SEM (*n* = 4 mice for Fc(mML1)B3, *n* = 3 mice for HGF). (B) Concentration of Fc and Fc(mML1)B3 (5.0 mg/kg) after 24 h (*n* = 3, *n* = 3) and 120 h (*n* = 3, *n* = 4) of SC injection in serum and liver lysate. The results are shown as the mean ± SEM. (C) Met activation *in vivo*. Mice were injected intravenously with either Fc (4.55 mg/kg, *n* = 1), Fc(mML1)B3 (5 mg/kg, *n* = 1), recombinant human HGF (0.5 mg/kg, *n* = 1), or PBS (*n* = 1). After 10 min, mice were euthanized, and the liver was collected. Representative images of hepatic tissue stained with an anti-phospho-Met antibody (green) are shown. Scale bar 100 μm. (D) Positive cells for phospho-Met were quantified from 5 random ×20 fields; the results were shown as the mean ± SEM. ns = not significant. ∗∗∗*p* < 0.001.

We assessed the Fc(mML1)B3 half-life in the circulation and if it reached the liver of mice. The long half-lives of Fc and antibodies are maintained primarily by binding to the neonatal Fc receptor (FcRn), which rescues Fc and antibodies from endosomal degradation and releases them into circulation.^37^^,^^38^ To keep the binding to the FcRn, we selected grafting loop locations distal to the FcRn binding site.^10^ As expected, the analysis of serum concentrations of Fc(mML1)B3 after a single subcutaneous injection at 5 mg/kg showed a long half-life (t_1/2_ = 163 h) (Figure 5A). Serum concentrations of Fc(mML1)B3 remained above 10 nM, sufficient to activate Met and downstream Akt and Erk1/2 (Figure 5A) for up to 14 days (Figure 5A). In contrast, HGF decreased to 0.01 nM within one day, a level that failed to activate Met (Figure 5A). The bioavailability of HGF after subcutaneous injection was much lower than that after intravenous injection (Figure 5A). This difference is likely due to its high molecular weight and heparin-binding properties, which can cause association with the extracellular matrix.^39^

We confirmed the presence of Fc(mML1)B3 in the liver at 24 h and 120 h after a subcutaneous injection of 5.0 mg/kg (24 h = 31.7 nM, 120 h = 22.9 nM in lysates) (Figure 5B). The concentration of Fc(mML1)B3 in the hepatic extracellular space available for Met receptors is likely higher than estimated because the liver lysate is mainly composed of cells. To examine Met phosphorylation *in vivo*, we administered a single intravenous dose of either Fc(mML1)B3 (5 mg/kg), Fc (4.55 mg/kg), HGF (0.5 mg/kg) as a positive control, or phosphate-buffered saline (PBS) as a negative control. Using immunofluorescence imaging, we observed Met activation in the HGF and Fc(mML1)B3 groups, with no significant difference in quantification (*p =* 0.24) (Figures 5C and 5D). These results indicated that Fc(mML1)B3 is bioactive with significantly improved circulation half-life and subcutaneous bioavailability compared to HGF.

### An lasso-graft-based hepatocyte growth factor mimetic ameliorated hepatic features of non-alcoholic steatohepatitis in a mouse model

**Figure 6:**
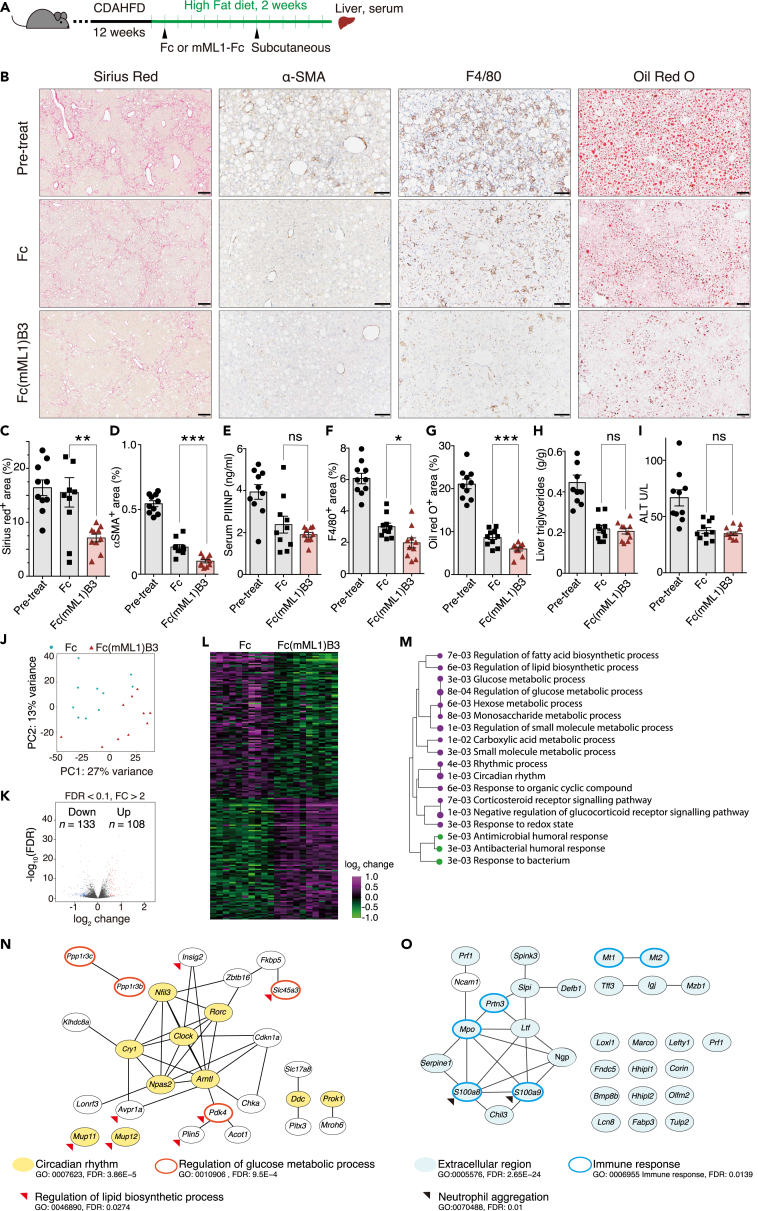
Fc(mML1)B3 subcutaneous injection under HFD conditions reduced hepatic lipid accumulation, macrophage infiltration, and fibrosis in the murine NASH model (A) After 12 weeks of feeding with a choline-deficient, L-amino acid-defined, high-fat diet (CDAHFD), male C57BL/6 mice were divided into three groups. One group served as a reference before treatment (Pretreatment group, *n* = 10). The other two groups received a high-fat diet (HFD) for 2 weeks and subcutaneous injection of 5 mg/kg of Fc(mML1)B3 (*n* = 10) or 4.3 mg/kg of Fc (*n* = 10) as a control once a week. (B) Representative images of hepatic tissue before treatment (pretreatment group) and after Fc(mML1)B3 or Fc administration. Paraffin sections of liver tissue were stained with Sirius red to detect collagen deposition (red) as a marker of fibrosis, labeled by immunohistochemistry for α-SMA as a marker of stellate cells (brown), F4/80 as a marker of macrophages (brown), or with Oil Red O for lipid deposition (red droplets). Scale bars: Sirius red and Oil Red = 200 mm, α-SMA and F4/80 = 100 mm. (C, D, F, G) Quantitative analysis of areas positive for Sirius red (C), α-SMA (D), F4/80 (F), and Oil Red O (G) staining in liver sections. (E) Serum procollagen type III N-terminal propeptide (PIIINP) levels. (H) Intrahepatic triglyceride levels. (I) Serum ALT levels. Data are presented as the mean ± SEM. (J–O) RNA sequencing analysis of liver tissues. (J) Principal component analysis showing clustering of two groups. (K) Volcano plot depicting the −log_10_ of the false discovery rate (FDR) and the log_2_-fold change (LFC) showing 108 upregulated and 133 downregulated genes in the Fc(mML1)B3 group. *p*_adj_ < 0.1 and fold change 2 were taken to indicate statistical significance. (L) Heatmap of differentially expressed genes. (M) Enriched pathways according to Gene Ontology analysis: magenta, upregulated; green, downregulated. Data are presented as −log_10_ of *p*-value (*p*_adj_ < 0.05). ns = not significant. ∗*p* < 0.05, ∗∗*p* < 0.01, ∗∗∗*p* < 0.001. (N, O) Interaction networks of relevant (N) upregulated genes and (O) downregulated genes in the Fc(mML1)B3 group vs. Fc group. Interaction networks were built from the STRING website and visualized in Cytoscape. See also Figures S3, S4, S6, and S7.

Given that recombinant HGF has shown positive effects on reducing fibrosis in various animal disease models,^23^^,^^24^^,^^25^^,^^30^^,^^32^^,^^33^^,^^40^ we assessed the therapeutic capabilities of Fc(mML1)B3 in a mouse model exhibiting hepatic features of NASH. Considering that advanced fibrosis stages can lead to fatal consequences for individuals with NASH,^14^^,^^16^^,^^41^ we selected a CDAHFD model known for inducing more severe fibrotic features compared to other rodent NASH models.^34^ Inbred C57BL/6J mice (n = 30) were fed a CDAHFD to induce NASH with fibrotic characteristics for 12 weeks. As a reference before treatment, one group of mice was sacrificed after the 12-week CDAHFD feeding period (Pretreatment group, *n* = 10). In this model, choline deficiency impairs the production of very low-density lipoproteins (VLDLs), essential for triglyceride excretion from the liver.^34^ Therefore, during the dosing period of Fc(mML1)B3, we switched to a high-fat diet (HFD) containing normal choline levels to enable VLDL production, which is also a possible mechanism of action of Met agonists.^29^^,^^42^ Mice fed the HFD were treated with either 5 mg/kg of Fc(mML1)B3 (n = 10) or 4.3 mg/kg of Fc (n = 10) as a control once a week for two weeks (Figure 6A).

Mice treated with Fc(mML1)B3 exhibited significantly lower collagen deposition in the liver parenchyma compared to both the Pretreatment group (*p* = 1.9E-05) and the Fc-treated group (*p* = 0.007) (Figures 6B and 6C). In contrast, the group treated with Fc showed a nonsignificant improvement trend (*p* = 0.78) (Figures 6B and 6C). Stellate cells contributing to fibrosis were significantly reduced in both groups (Pretreatment vs. Fc(mML1)B3: *p* = 1,7E−12*;* Pretreatment vs. Fc: *p* = 1.8E−09), but the effect was more substantial in the Fc(mML1)B3 group (*p* = 0.0001), as indicated by staining of the stellate cell marker α-smooth muscle actin (α-SMA) (Figures 6B and 6D). The serum concentrations of procollagen type III N-terminal propeptide (PIIINP),^43^ a fibrosis marker, were significantly decreased in both groups (Pretreatment vs. Fc: *p* = 0.009; Pretreatment vs. Fc(mML1)B3: *p* = 2.8E−05), but the decreasing trend was greater in the Fc(mML1)B3 group (*p* = 0.26) (Figure 6E). These results suggest that Fc(mML1)B3 treatment improves fibrosis compared to Fc treatment, as evidenced by a significant reduction in collagen deposition in the Sirius Red staining.

Macrophage infiltration in the liver parenchyma, driven by lipid accumulation, is associated with the development of NASH.^44^^,^^45^ We observed a significantly lower positive area of the F4/80 macrophage marker in the Fc(mML1)B3 group (*p =* 0.016) than in the Fc group. Notably, there was also a significant improvement in the Fc-treated group (Pretreatment vs. Fc(mML1)B3: *p* = 4,7E−08*;* Pretreatment vs. Fc: *p* = 4.9E−07) (Figures 6B and 6F). However, there was no clear evidence of an anti-inflammatory effect of Fc(mML1)B3 under the HFD condition on the hepatic cytokine profile at the endpoint of the treatment (protein levels: TNF- α (*p =* 0.55), IL-6 (*p =* 0.25), TGF-β (*p =* 0.42) and CCL5 (*p =* 0.069); mRNA expression: *CXCL1* (*p* = 0.99), *CCL2* (*p* = 0.42), *TGF-ß* (*p* = 0.19) and *CCL5* (*p* = 0.55; Figure S3). Under the standard diet (later discussed), the mRNA expression of *CXCL1* (*p* = 0.04) and *CCL2* (*p* = 0.002) showed lower levels in the Fc(mML1)B3 group (Figure S3).

Next, we assessed hepatic lipid accumulation through oil red staining (Figures 6B and 6G). The Fc(mML1)B3 group exhibited significantly lower lipid accumulation than the Fc group (*p* = 0.002). However, there were no differences in hepatic triglycerides measured after tissue-lipid extraction between the Fc(mML1)B3 and Fc-treated groups (*p* = 0.62) (Figure 6H). These results may be associated with the accumulation of different lipids besides triglycerides, including cholesterol esters and other lipotoxic lipids (*i.e*., excess free cholesterol), in the hepatic lipid droplets observed in NASH.^46^ The improvement in alanine aminotransferase (ALT) level, a marker of hepatocyte damage in serum, did not differ between both groups at this time point (*p* = 0.37) (Figure 6I). However, it became significantly lower in the Fc(mML1)B3-treated group after four weeks in another group of mice that continued the treatment period up to 4 weeks under high-fat diet (*p* = 0.0007) (5 mg/kg once a week) (Figure S4).

**Figure 7:**
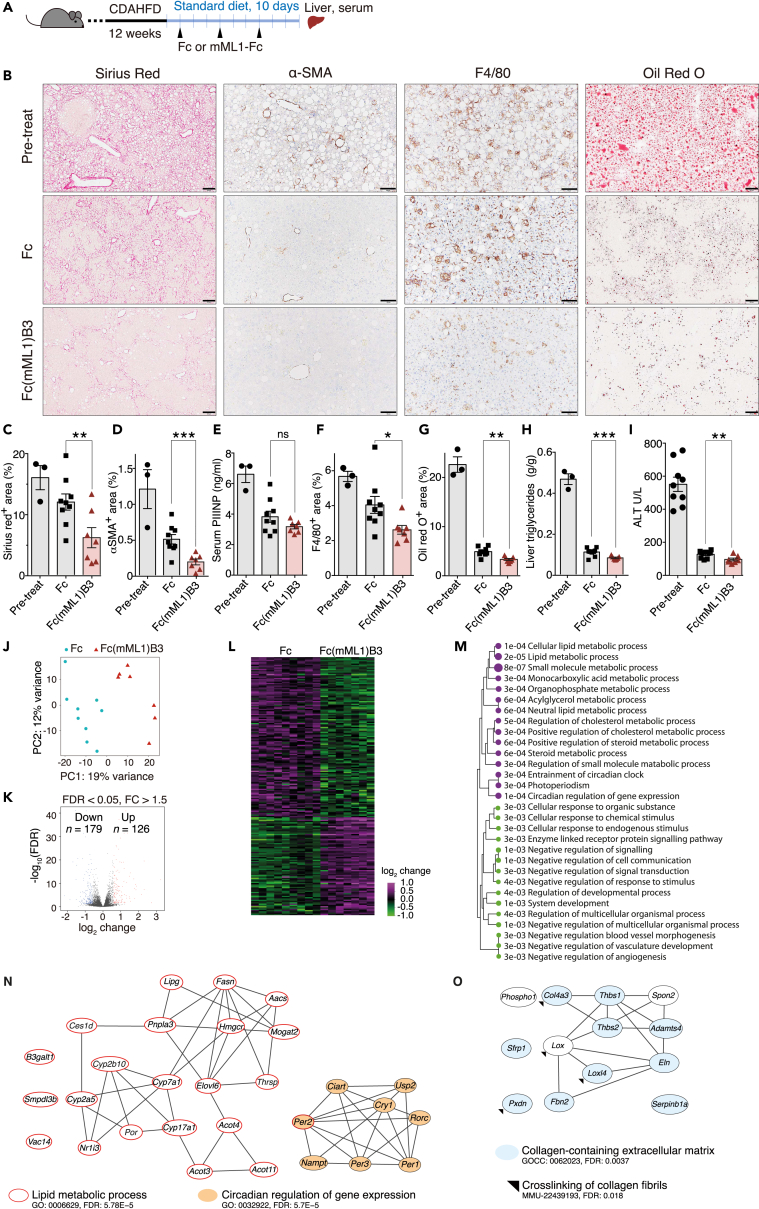
Subcutaneous injection of Fc(mML1)B3 with feeding standard diet reduced features of NASH (A) After 12 weeks of feeding with the CDAHFD, male C57BL/6 mice were divided into three groups. One group served as a reference before treatment (Pretreatment group, *n* = 3). The other two groups received a standard diet for 10 days and subcutaneous injection of either 5 mg/kg of Fc(mML1)B3 (*n* = 7) or 4.3 mg/kg of Fc (*n* = 9) as control every 3 days. (B) Representative images of hepatic tissue before treatment (Pretreatment group) and after Fc(mML1)B3 or Fc administration. Paraffin sections of liver tissue were stained with Sirius red to detect collagen deposition (red) as a marker of fibrosis, labeled by immunohistochemistry for α-SMA as a marker of stellate cells (brown) , F4/80 as a marker of macrophages (brown), or with Oil Red O for lipid deposition (red droplets). Scale bars: Sirius red and Oil Red = 200 mm, α-SMA and F4/80 = 100 mm. (C, D, F, G) Quantitative analysis of areas positive for Sirius red (C), α-SMA (D), F4/80 (F), and Oil Red O (G) staining in liver sections. (E) Serum procollagen type III N-terminal propeptide (PIIINP) levels. (H) Intrahepatic triglyceride levels. (I) Serum ALT levels. Data are presented as the mean ± SEM. (J–M) RNA sequencing analysis of liver tissues. (J) Principal component analysis showing clustering of two groups. (K) Volcano plot depicting the −log_10_ of the false discovery rate (FDR) and the log_2_-fold change (LFC) showing 126 upregulated and 179 downregulated genes in the Fc(mML1)B3 group. *p*_adj_ < 0.05 and fold change 1.5 were taken to indicate statistical significance. (L) Heatmap of differentially expressed genes. (M) Enriched pathways according to Gene Ontology analysis: magenta, upregulated; green, downregulated. Data are presented as −log_10_ of *p*-value (*p*_adj_ < 0.05). ns = not significant. ∗*p* < 0.05, ∗∗*p* < 0.01, ∗∗∗*p* < 0.001. (N, O) Interaction network of relevant upregulated genes (N) and downregulated genes (O) in the Fc(mML1)B3 group vs. Fc group. See also Figure S8.

HFD increases hepatic triglyceride content, which contributes to the progression of NAFLD,^47^ and dietary changes improve NASH.^48^ Therefore, we also performed a similar experiment in mice fed CDAHFD for 12 weeks and then gave them a standard diet and three subcutaneous doses of either 5 mg/kg Fc(mML1)B3 (*n* = 7) or 4.3 mg/kg Fc (*n* = 9) once every three days for ten days (Figure 7A). Under the standard diet, both groups showed improvement in the hepatic features developed after the CDAHFD period, with all parameters significantly lower in the Fc(mML1)B3-treated group (Figures 7B–7I), including collagen deposition (Fc(mML1)B3 vs. Fc: *p* = 0.01, Pretreatment vs. Fc: *p* = 0.15, Pretreatment vs. Fc(mML1)B3: *p* = 0.007), stellate cells (*p* = 1.4E-10), and macrophage-positive areas (*p* = 0.03), lipid accumulation (*p* = 0.001), hepatic triglycerides (*p* = 0.003), and serum ALT (*p* = 0.005). Except for the deposition of collagen fibers, the Fc-treated groups also exhibited the amelioration of other features. This improvement could be explained by the liver’s self-renewal capacity and choline availability even when these mice received a high-fat diet during treatment.

In addition, we performed RNA sequencing analysis of liver tissues from mice treated with Fc(mML1)B3 and Fc. The mice fed the HFD during treatment showed the downregulation of 133 genes and the upregulation of 108 genes in the Fc(mML1)B3 group (Figures 6J–6L, FDR <0.1 and FC > 2). Pathway analysis and protein-protein interaction network analysis showed enrichment for upregulated Gene Ontology (GO) terms mainly related to the regulation of a variety of metabolic pathways, including lipid and glucose metabolic regulation and circadian rhythm. (Figures 6M and 6N). Inflammation-related terms were also downregulated in the Fc(mML1)B3 group, specifically neutrophil-related genes known to contribute to the development of NASH, such as myeloperoxidase (*MPO*) and *S100A8/A9*^49^ (Figures 6M and 6O), suggesting the improvement of liver metabolism and suppression of inflammation in the Fc(mML1)B3 group. We also observed the downregulation of *lysyl oxidase-like 1* (*Loxl1*) in the Fc(mML1)B3 (Figure 6O). Loxl1 is a member of the lysyl oxidases (LOX) family associated with elastin crosslinking, a critical factor that limits fibrosis improvement and has been reported to accumulate at the cirrhotic stage.^50^ Another downregulated gene that drew our attention was *Serpine1* (Figure 6O), which encodes plasminogen activator inhibitor type-1 (PAI-1), known to have an antifibrinolytic effect.^51^

Mice that were fed the standard diet during treatment showed 179 downregulated genes and 126 upregulated genes in the Fc(mML1)B3-treated group (Figures 7J–7L, FDR <0.1 and FC > 2). Pathway enrichment and protein-protein interaction network analyses revealed enriched upregulated terms related to the regulation of lipid and glucose metabolism (Figures 7M and 7N). In contrast, terms associated with extracellular matrix and collagen formation were downregulated. These downregulated genes include the following: *Lox* and *lysyl oxidase-like 4 (Loxl4)* proposed as a critical determinant in lung fibrosis^52^; *Collagen type IV alpha 3 chain* (*Col4α3)* upregulated in a mouse liver fibrosis model^53^; *Thrombospondin-1* (*Thbs1*) upregulated in NAFLD and liver fibrosis models, where it plays a role by activating TGF-β and also by inducing angiogenesis and sinusoidal remodeling^54^^,^^55^; *Thrombospondin-2* (*Thbs2*) proposed as a biomarker of advanced fibrosis in NAFLD^56^; *Elastin* (*Eln*), the most stable protein of the extracellular matrix (ECM) observed in cirrhotic livers^50^ and correlated with the development of hepatocellular carcinoma^57^ (Figure 7O).

Taken together, these results showed that Fc(mML1)B3 ameliorates the deleterious effects induced by CDAHFD in the liver, including collagen deposition, inflammation, and lipid accumulation, and these effects may be potentiated by diet modifications.

**Figure 8:**
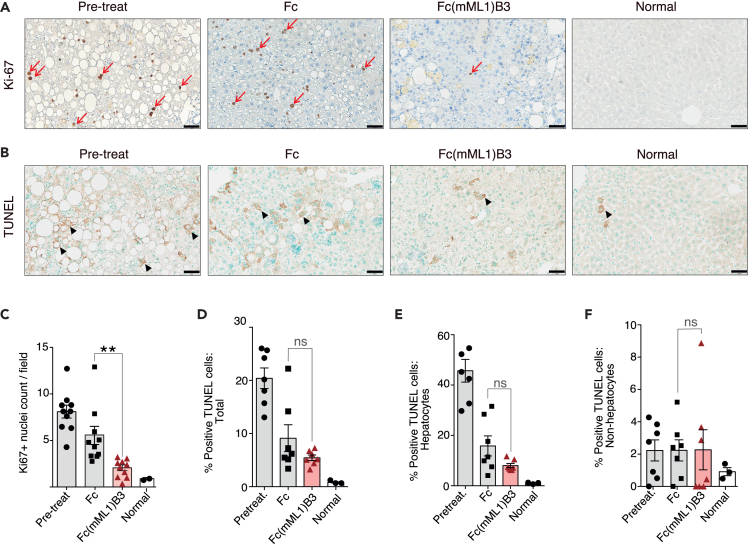
Reduction of hepatocyte proliferation and apoptosis by Fc(mML1)B3 in the murine NASH model (A) Representative immunohistochemistry images for Ki-67 as a marker of proliferative cells. Arrows indicate Ki-67 positive nuclei. Scale bars: 50 μm. (B) Representative immunohistochemistry images of TUNEL as a marker of apoptosis. Black arrows indicate TUNEL positive hepatocytes. Scale bars: 50 μm. (C) Quantification of Ki-67 positive nuclei per field. (D) Quantification of apoptotic hepatic cells (total). (E) Quantification of apoptotic hepatocytes. (F) Quantification of apoptotic hepatic cells excluding hepatocytes. Data are presented as the mean ± SEM. ns = not significant. ∗∗*p* < 0.01. See also Figure S5.

Additionally, we evaluated the proliferation (Figure 8A) and apoptosis (Figure 8B) of hepatocytes at pretreatment and the final stage of treatment in the HFD condition. The pretreatment group showed the highest hepatocyte proliferation compared to the Fc and Fc(mML1)B3 groups, consistent with the idea that liver proliferation contributes to fibrosis development in NAFLD mouse models.^58^ Notably, the Fc(mML1)B3 group exhibited lower hepatocyte proliferation compared to Fc (*p* = 0.003). (Figures 8A and 8C). Similarly, the percentage of apoptotic cells in the liver was higher in the pre-treatment group, but decreased in the Fc group and the Fc(mML1)B3 group, with a more significant difference observed in the Fc(mML1)B3 group (Figure 8D, Pre-treatment vs. Fc: *p* = 0.004, Pre-treatment vs. Fc(mML1)B3: *p* = 7.2E-06). Apoptosis mainly occurred in hepatocytes, and there was no significant difference in the percentage of TUNEL-positive cells excluding hepatocytes (Figures 8E and 8F). We also performed an immunofluorescent version of the TUNEL assay, which allows a better definition of the positive cells. We found that the number of apoptotic bodies was higher in the pretreatment group compared to the Fc and Fc(mML1)B3 groups. Notably, the Fc(mML1)B3 group exhibited lower hepatic apoptosis compared to Fc (*p* = 0.001) (Figure S5). These results suggest that Fc(mML1)B3 promotes liver recovery, as evidenced by reduced hepatocyte proliferation and apoptosis.

Finally, no clinical signs, macroscopic, or microscopic abnormalities were observed in mice’s liver, spleen, kidneys, and lungs after two weeks of subcutaneous Fc(mML1)B3 administration (Figure S6).

## Discussion

In this study, we successfully designed an HGF mimetic by incorporating RaPID-identified mMet-binding peptides into optimized Fc loops. The Fc(mML1)B3 demonstrates an extended biological half-life of up to 163 h when administered subcutaneously once a week; it also showed accumulation in the liver and phosphorylation of the Met receptor *in vitro* and *in vivo*. Moreover, it exhibits significant improvements in NASH-related liver features, decreased apoptosis, and reduced fibrosis in a model of severe fibrosis. These advancements address the limitations associated with recombinant HGF, such as its short half-life and low subcutaneous bioavailability, thereby eliminating the need for frequent intravenous administration in animal models.^25^^,^^26^^,^^27^^,^^29^^,^^33^^,^^40^

Liver fibrosis is generally considered reversible when the underlying stimuli are removed.^13^ However, the reversibility of liver cirrhosis and its systemic effects, such as portal hypertension, is not well understood. Irreversible liver fibrosis is caused by non-reductive crosslinking of elastin and collagen fibers,^17^ a process mediated by proteins of the LOX family.^59^ This irreversible fibrosis is strongly linked with liver cirrhosis and hepatocellular carcinoma.^57^ Our RNA-seq data showed that, under standard diet or high-fat diet conditions, Fc(mML1)B3 suppressed the expression of genes related to this crosslinking, such as *Eln*, *Lox*, *Loxl4*, and *Loxl1*. Furthermore, α-SMA-positive cells in the liver, namely activated hepatic stellate cells, which play a crucial role in fibrosis associated with NASH,^13^ decreased under both diet conditions after treatment with both Fc(mML1)B3 and Fc, with a more pronounced decrease in the Fc(mML1)B3 group. Additionally, the administration of Fc(mML1)B3 resulted in a significant reduction in liver collagen fibers. Based on these findings, we conclude that Fc(mML1)B3 exerts anti-fibrotic effects by regulating ECM components. This anti-fibrotic action could be effective even in the advanced stages of fibrosis due to the involvement of elastin crosslinking-related genes. This potential should be analyzed in future experiments using a liver cirrhosis model.

We observed a notable reduction in the F4/80-positive area in mice liver treated with Fc(mML1)B3. The expansion of the macrophage area in the liver has previously been linked to changes in shape and distribution resulting from abnormal lipid accumulation.^44^^,^^60^ For instance, in conditions such as steatosis, macrophages tend to aggregate, while in atherosclerotic plaques, they exhibit a foam cell-like morphology, enlarging in size due to increased lipid uptake.^60^^,^^61^ We suggest that the decrease in the F4/80-positive macrophage area induced by Fc(mML1)B3 indicates a restoration of the distribution and characteristics of normal liver macrophages. The significant reduction in liver lipid droplets caused by Fc(mML1)B3 appears to facilitate the improved systemic movement of lipids from the liver; this is further supported by the upregulation of genes associated with terms such as "lipid metabolism process" and "regulation of lipid biosynthetic process." Consequently, the reduction in hepatic steatosis leads to the secondary observation of the recovery of normal liver macrophage distribution and characteristics. This collective evidence suggests that Fc(mML1)B3 contributes to restoring typical liver macrophage features by mitigating liver lipid accumulation, as indicated by histological changes and gene expression patterns related to lipid metabolism.

One potential concern when using HGF agonists as therapeutic agents is their impact on the cardiovascular system,^25^ such as the increased risk of endothelial deposition and arteriosclerosis due to the mobilization of lipids from the liver into the bloodstream. In our model, the administration of Fc(mML1)B3 did not increase serum triglycerides, and mice increased their body weight to the control group’s average (Figures S7 and S8). The weight gain suggests the transport of accumulated liver lipids to safe storage locations, such as adipose tissue. While an increase in circulating triglycerides was not observed, the possibility of a temporary elevation cannot be ruled out, necessitating long-term experiments to assess the risk of hyperlipidemia and cardiovascular diseases. Another potential concern is carcinogenesis due to its ability to induce cell proliferation, for example, in hepatocellular carcinoma.^62^^,^^63^^,^^64^ Although we did not observe enhanced proliferation of hepatocytes or cancer development by Fc(mML1)B3 at the endpoint of treatment, a 2-week duration may be too short to confirm this, and more extended treatment periods should be performed.

The therapeutic potential of HGF has been demonstrated in preclinical models of several diseases, including NASH.^21^^,^^23^^,^^25^^,^^26^^,^^27^^,^^28^ Our approach to exploring the HGF/Met potential involved Met agonists based on Fc of IgG with peptide sequences binding to Met receptor. Recently, a similar approach was proposed by generating mouse monoclonal antibodies against the extracellular domain of the human Met. This antibody was tested in a NASH model using mice that underwent the transplantation of human hepatocytes. Their results showed the inhibition of inflammatory cell infiltration, amelioration of fibrosis, induction of the proliferation of human hepatocytes, and the restoration of the HGF-Met axis, which they reported to be blocked in human NASH.^65^ These findings align with our results and encourage further experiments with agonists that include the Fc fraction in their structure to overcome the limitations of HGF. However, experiments in non-human primates may be necessary to create a closer model of human NASH that is more translatable to the clinic, including systemic changes.

FcRn-mediated transport not only extends the half-life of the Fc-coupled biologics but also enables less invasive administration routes such as intranasal and inhaled delivery.^37^^,^^38^ Human nasal and alveolar epithelial cells express FcRn and are capable of transcytosing Fc-containing molecules.^66^^,^^67^^,^^68^ By leveraging this characteristic, the nasal and inhalation routes could potentially be utilized to deliver Fc-based growth factor mimetics.

While this work primarily focuses on the therapeutic potential of Fc(mML1)B3 in the hepatic manifestations of NASH, LG-based HGF mimetics offer promise as a treatment option for several diseases that have been identified as potential targets for recombinant HGF, including amyotrophic lateral sclerosis.^20^ In the context of central nervous system disorders, our approach enables the production of HGF mimetics capable of crossing the blood-brain barrier by utilizing an anti-transferrin receptor antibody as a scaffold for LG.^10^ Furthermore, the RaPID system, which can generate macrocyclic peptides targeting diverse receptors,^10^^,^^11^^,^^12^^,^^36^^,^^69^ opens up the possibility of applying this strategy to other dimeric cell surface receptor pairs such as receptor tyrosine kinase families and transforming growth factor receptor families. The LG of immunoglobulin Fc facilitates the design of growth factor and cytokine mimetics with favorable pharmacokinetics and stability, thereby broadening their therapeutic applications.

### Limitations of the study

A limitation of this study is that due to the choline deficiency-related VLDL formation impairment, the CDAHFD-induced NASH model rapidly shows severe fibrosis and steatosis with poor body weight gain. This disease progression does not entirely resemble the chronic and systemic nature of NASH in humans.^34^^,^^70^^,^^71^ Therefore, it was not possible to assess features such as adipose tissue gain and peripheral insulin sensitivity. Further studies are required to evaluate these aspects in other models. Indeed, mice fed CDAHFD had reduced body weight gain compared to those fed a standard diet but quickly returned to normal levels when choline was supplied, regardless of the fat content in the diet (Figures 7 and 8). The choline itself may also have a therapeutic effect,^72^ and NASH can show some improvement with dietary changes.^48^^,^^73^ This diet effect could explain why, in our models, the Fc-treated groups ameliorated hepatic features of NASH while fed a standard diet and HFD, although to a lesser extent than the Fc(mML1)B3-treated groups.

## STAR★Methods

### Key resources table

**Table:** 

REAGENT or RESOURCE	SOURCE	IDENTIFIER
**Antibodies**		

Anti-phospho-Met (Tyr1234/1235) (D26) anti-mouse	Cell Signaling	mAb #3077, RRID: AB_2143884
Polyclonal Goat Anti-Rabbit Immunoglobulins/HRP	Dako/Agilent	P044801-2
Anti-PA tag antibody NZ-1	Fujifilm	012-25863, RRID: AB_3094664
Alexa Fluor 488-labeled goat anti-human IgG	Thermo Fisher	A11013, RRID: AB_2534080
Phospho-pErk1/2 (T202/Y204) (D13.14.4E) anti-mouse	Cell Signaling	mAb #4370, RRID: AB_2315112
p44/42 (Erk1/2) (137F5) anti-mouse	Cell Signaling	mAb #4695, RRID: AB_390779
Phospho-Akt (S473) (D9E) anti-mouse	Cell Signaling	mAb #4060, RRID: AB_2315049
Akt (pan) (11E7) anti-mouse	Cell Signaling	mAb #4685, RRID: AB_2225340
GAPDH (14C10) anti-mouse	Cell Signaling	mAb #2118, RRID: AB_561053
anti-Met antibody (B-2)	Santa Cruz	Sc-8057, RRID: AB_673755
Met (25H2)	Cell Signaling	mAb #3127, RRID: AB_331361
Rabbit polyclonal anti-human HGF antibody	Tahira et al.^74^	N/A
Biotinylated-rabbit anti-human HGF antibody	Tahira et al.^74^	N/A
Pierce Streptavidin conjugated with HRP	Thermo Fisher	21130
Anti-Met (c-Met) (phospho Y1230 + Y1234 + Y1235)	Abcam	ab5662, RRID: AB_305029
Goat Anti-Rabbit IgG H&L (Alexa Fluor® 488)	Abcam	ab150077, RRID: AB_2630356
Rat monoclonal F4/80 (A3-1)	Bio-Rad	MCA497RT, RRID: AB_1102558
Anti-alpha smooth muscle Actin	Abcam	ab5694, RRID: AB_2223021
Anti-Ki67	Abcam	ab16667, RRID: AB_302459

**Biological samples**		

Mouse liver	This study	N/A
Mouse serum	This study	N/A
Mouse lung	This study	N/A
Mouse spleen	This study	N/A
Mouse kidney	This study	N/A

**Chemicals, peptides, and recombinant proteins**		

mouse Met ectodomain fused with Fc	R&D systems	527-ME
Human Recombinant HGF	Kringle Pharma	N/A
Fc	This Study	N/A
Fc(mML1)T1	This Study	N/A
Fc(mML1)T2	This Study	N/A
Fc(mML1)T3	This Study	N/A
Fc(mML1)M2	This Study	N/A
Fc(mML1)M3	This Study	N/A
Fc(mML1)B1	This Study	N/A
Fc(mML1)B2	This Study	N/A
Fc(mML1)B3	This Study	N/A

**Critical commercial assays**		

Expi293 expression system	Thermo Fisher	A14525
HiTrap Protein A HP column	Cytiva	29048576
Superdex 200 Increase 10/300 GL column	Cytiva	28990944
Proteus NoEndo Spin Column Kit	Protein Ark	GEN-NoE24Micro
ToxinSensor Chromogenic LAL Endotoxin Assay Kit	GenScript	L00350
Bicinchoninic acid assay	Thermo Fisher	23225
Lysis Buffer	RayBiotech	AA-LYS
Mouse RTK phosphorylation Antibody Array C1 Kit	RayBiotech	AAM-PRTK-1-2
3-(4,5-dimethylthiazol-2-yl)-5-(3-carboxymethoxyphenyl)-2-(4-sulfophenyl)-2H-tetrazolium, inner salt (MTS) assay	Promega	G5421
human IgG ELISA quantitation set	Bethyl Laboratories	E80-104-34
RNase H minus reverse transcriptase	Promega	M5301
Dynabeads protein G	Thermo Fisher	10003D
Picrosirius Red Stain Kit	Polyscience	24901
Oil Red O	Nacalai-Tesque	25633-92
Mayer's Hematoxylin	Fujifilm	131-09665
0.1% Eosin Y, Ethanol Solution	Fujifilm	054-06505
Histofine® Simple Stain MAX PO	Nichirei	414131F
CF™594 TUNEL Assay	Biotium	30064
TUNEL Assay Kit-HRP-DAB	Abcam	ab206386
Mouse N-Terminal Procollagen III Propeptide (PIIICNP)	Cusabio	CSB-E13334m
ELISA MAX™ Standard Set Mouse TNF-α	Biolegend	430901
RANTES Mouse ELISA Kit	Thermo Fisher	KMC1031
TGF beta-1 Human/Mouse Uncoated ELISA Kit	Thermo Fisher	88-8350-88
IL-6 Mouse Uncoated ELISA Kit	Thermo Fisher	88-7064-88
LabAssay™Triglyceride	Fujifilm	632-50991
Alanine Aminotransferase Activity Assay Kit	Sigma-Aldrich	MAK052
RNA later ™ Stabilization Solution	Thermo Fisher	AM7020
QIAzol Lysis Reagent	QIAGEN	79306
SuperScript™ III Reverse Transcriptase	Thermo Fisher	18080093

**Deposited data**		

RNA sequencing data (GEO depository)	This study	GSE233051
Source data (Mendeley Dataset)	This study	https://doi.org/10.17632/sympm7wch5.1

**Experimental models: Cell lines**		

AML12 immortalized mouse hepatocytes	ATCC	CRL-2254
HEK293T	ATCC	CRL-11268

**Experimental models: Organisms/strains**		

C57BL/6 female mice	SLC	N/A
C57BL/6 male mice	SLC	N/A

**Oligonucleotides**		

Taqman gene expression assay: Tgfb1 (2146278 C3)	Thermo Fisher	Mm01178820_m1
Taqman gene expression assay: Ccl2 (2095828 F6)	Thermo Fisher	Mm00441243_g1
Taqman gene expression assay: Ccl5 (2131122 F1)	Thermo Fisher	Mm01302428_m1
Taqman gene expression assay: Cxcl1 (2143422 G7)	Thermo Fisher	Mm04207460_m1
Taqman gene expression assay: Mmp13 (2136171 D9)	Thermo Fisher	Mm00439491_m1
Taqman gene expression assay: Actb (2152555 B7)	Thermo Fisher	Mm02619580_g1

**Recombinant DNA**		

pcDNA3.1-based backbone	N/A	https://www.genomics-online.com/vector-backbone/55/pcdna3.1/
cDNA libraries	Bioengineering Lab Co., Ltd	N/A

**Software and algorithms**		

FlowJo software	BD Biosciences	https://www.flowjo.com/
Image J software	NIH (National Institutes of Health)	https://imagej.net/
Immunohistochemistry Image Analysis Toolbox	Shu et al.^75^	https://imagej.net/ij/plugins/ihc-toolbox/
Fastp	Chen et al.^76^	https://github.com/OpenGene/fastp
Bowtie2 (ver 2.4.5).	Langmead et al.^77^	https://bowtie-bio.sourceforge.net/bowtie2/index.shtml
STAR tool (ver 2.7.10a)	Dobin et al.^78^	https://github.com/alexdobin/STAR
featureCounts in the Subread package (ver. 2.0.3).	Liao et al.^79^	https://github.com/ShiLab-Bioinformatics/subread
iDEP 0.951	Ge et al.^80^	https://idep.diatomicsbase.bio.ens.psl.eu/
DESeq2	Love et al.^81^	https://bioconductor.org/packages/release/bioc/html/DESeq2.html
String online database (ver 11.5)	Szklarczyk et al.^82^	https://string-db.org/
Cytoscape (version 3.9.1)	Shannon et al.^83^	https://cytoscape.org/
StringApp (version 2.0.1)	Doncheva et al.^84^	https://apps.cytoscape.org/apps/stringapp
GraphPad Prism (ver 6.0days)	Dotmatics	https://www.graphpad.com/support/prism-6-updates/

**Other**		

FIT system	Goto et al.^85^	N/A
RaPID approach	Passioura and Suga^86^	N/A
MiSeq next-generation sequencer	Illumina	SY-410-1003
EC800 system Flow Cytometry Analyzer	Sony	https://www.sonybiotechnology.com/wordpress/wp-content/uploads/2016/05/eclipse_brochure.pdf
Fusion Solo S chemiluminescence imaging system	Vilber Lourmat	https://www.vilber.com/fusion-solo-s/
Luminescent Image Analyzer LAS-3000 mini Ver. 2.2	Fujifilm	N/A
Precellys evolution touch homogenizer	Bertin Technologies	P002511-PEVT0-A.0
Tissue-Tek O.C.T embedding medium	Sakura Finetek	4583
Keyence BZ-800 microscope	Keyence Corporation	https://www.keyence.com/ss/products/microscope/bz-x800_research/
Olympus microscope BX51	Olympus	https://www.olympus-lifescience.com/en/microscope-resource/primer/techniques/fluorescence/bx51fluorescence/
RX-860 Rotatory Microtome	Yamato	http://www.yamato-web.co.jp/products-html/RX-860.html
LEICA CM1950 Cryostat	Leica Biosystems	https://www.leicabiosystems.com/en-jp/histology-equipment/cryostats/leica-cm1950/
ARVO MX plate reader	Perkin Elmer	https://www.perkinelmer.co.jp/reader/tabid/144/Default.aspx
CDAHF diet (L-amino acid diet with 60 kcal% fat, 0.1% methionine, and no added choline)	Research Diets	A06071302
HFD (60 kcal% fat, and crystalline amino acids)	Research Diets	D06030601
RNA sequencing	Bioengineering Lab Co., Ltd.	https://gikenbio.com/corporate/
DNBSEQ-G400 sequencer	MGI Tech	https://en.mgi-tech.com/products/instruments_info/2/
MGIEasy RNA Directional Library Prep Set	MGI Tech	https://en.mgi-tech.com/products/reagents_info/14/
AATI Fragment Analyzer	Advanced Analytical Technologies	https://www.agbl.net/advanced-analytical-technologies-aati/
MGIEasy Circularization Kit	MGI Tech	https://en.mgi-tech.com/products/reagents_info/4/
DNBSEQ-G400RS High-throughput Sequencing Kit	MGI Tech	https://en.mgi-tech.com/products/reagents_info/20/
Quantitative RT-PCR using the ViiA™ 7 Real-Time PCR System	Thermo Fisher	https://www.thermofisher.com/jp/ja/home/life-science/pcr/real-time-pcr/real-time-pcr-instruments/viia-7-real-time-pcr-system.html

### Resource availability

#### Lead contact

Further information and requests for resources and reagents should be directed to and will be fulfilled by the Lead Contact, Katsuya Sakai (k_sakai@staff.kanazawa-u.ac.jp).

#### Materials availability

Reagents and plasmids generated in this study are available to the lead contact upon request.

#### Data and code availability

• The RNA sequencing data for this study are accessible at the GEO repository (NCBI) under accession number GSE233051. Original western blot images can be found in the supplementary section of the paper. Microscopy data mentioned in this article will be provided to the lead contact upon request. The source data are available in the Mendeley Dataset. https://doi.org/10.17632/sympm7wch5.1.
• This paper does not report the original code.
• The lead contact will share all data reported in this paper upon request.

### Experimental model and study participant details

#### Mouse models

5–8-week-old C57BL/6J inbred mice weighing 20-22 g were purchased from Japan SLC, Inc. They were housed in a specific-pathogen-free animal care facility at the Institute for Experimental Animals Kakuma Branch at Kanazawa University. Same-sex mice were randomly allocated in cages (2-4 mice per cage) at 23±3°C, humidity 55±10%, with a 12-hour light/12-hour dark cycle starting at 8:45 am. Animals had *ad libitum* access to water, and either chow diet or CDAHF diet (L-amino acid diet with 60 kcal% fat, 0.1% methionine, and no added choline) purchased from Research Diets, Inc. or high-fat diet (HFD, 60 kcal% fat, and crystalline amino acids, Research Diets), depending on the study performed. All experimental procedures were approved by the Committee on Animal Experimentation of Kanazawa University. Both male and female mice were included in the studies as indicated for each experiment, and all mice were immunocompetent and healthy before experimentation.

### Method details

#### Macrocyclic library design

A thioether-macrocyclic peptide library was constructed by using *N*-(2chloroacetyl)-D-tyrosine (ClAc^D^Y) or *N*-(2chloroacetyl)-L-tyrosine (ClAc^L^Y) as initiators in a FIT system reaction.^8^ The underlying mRNA library was designed to have an AUG (ClAc^D/L^Y) initiator codon followed by 4–15 NNK codons (N = G, C, A, or U; K = G or U), which code random proteinogenic amino acid residues, followed by a fixed UGC codon that assigns Cys. The theoretical diversity of macrocyclic peptides based on the quantitative assessment of the efficiencies of the individual transformation steps is ≥10^12^. After *in vitro* translation, a thioether bond spontaneously formed between the N-terminal ClAc group of the initiator D/L-Tyr residue and the sulfhydryl group of a downstream Cys residue to generate the macrocyclic peptide backbone.

#### Selection of macrocyclic peptides binding to mMet

Affinity selection was performed using the ^D^Y- or ^L^Y-initiated libraries against mouse Met ectodomain fused with Fc (R&D systems, Minneapolis, USA) by employing a RaPID approach.^8^ The mRNA library and ClAc-^D^Y-tRNA^fMet^_CAU_ or ClAc-^L^Y-tRNA^fMet^_CAU_ were prepared as reported.^8^^,^^35^ Briefly, 1 μM mRNA library was ligated to a puromycin-linked oligonucleotide (1.5 μM) using T4 RNA ligase at 25°C for 30 min. After purification by phenol-chloroform extraction and ethanol precipitation, 1.2 μM mRNA-puromycin conjugate was translated at 37°C for 30 min in a methionine-deficient FIT reaction containing 25 μM ClAc-^D^Y-tRNA^fMet^_CAU_ or ClAc-^L^Y-tRNA^fMet^_CAU_ to generate the peptide library. Following translation, incubation at 25°C for 12 min was performed to facilitate mRNA-peptide complexes, 2,2',2 ",2'"-(Ethane-1,2-diyldinitrilo) tetra-acetic acid (EDTA) was added to a final concentration of 20 mM and the reaction was incubated at 37°C for 30  min to remove the mRNA-peptide complexes from the ribosomes. The product was subsequently reverse transcribed using RNase H minus reverse transcriptase (Promega, USA) at 42°C for 1 h. The final library was counter-selected against Dynabeads protein G (Thermo Fisher Scientific, USA) to remove undesired bead binders. The counter-selection was repeated twice for the first round of selection and six times for all later rounds. For affinity selection to mMet, the peptide–mRNA/cDNA solution was incubated with 200 nM mouse Met ectodomain fused with Fc (R&D systems, USA) immobilized on Dynabeads protein G for 30 min at 4°C to isolate mMet-binders. The fused peptide–mRNA/cDNA was separated from the beads by incubating in 1× polymerase chain reaction (PCR) buffer heated for 5 min at 95°C, and the amount of eluted cDNAs was measured by quantitative PCR. The remaining cDNAs were amplified by PCR and then purified and transcribed to produce an enriched mRNA library for the next selection round. The final enriched cDNA was sequenced using a MiSeq next-generation sequencer (Illumina, San Diego, USA).

#### Protein design, construction, and purification

For the construction of the Fc protein inserted with mMet-binding macrocyclic peptides, human IgG1 Fc (residues 104-330, Uni-Prot P01857) was used without any tags. The internal sequence of mMet-binders appended with spacer residues of Gly-Gly-Ser at both ends was inserted into internal structural loops (B1 for initial selection, T1 to B3 for mML1). All expression constructs were made using a pcDNA3.1-based backbone with an appropriate signal peptide, and the coding region of all expression constructs was verified by DNA sequencing. Protein expressions were performed using the Expi293 expression system (Thermo Fisher Scientific, USA). Secreted proteins were purified on a HiTrap Protein A HP column (Cytiva, USA) using elution with 0.1 M glycine-HCl buffer (pH 3.2), followed by neutralization, dialysis against phosphate-buffered saline (PBS), and size-exclusion chromatography on a Superdex 200 Increase 10/300 GL column (Cytiva, USA) equilibrated with PBS. To treat the mouse NASH model, Fc, and Fc(mML1)B3 proteins were subjected to endotoxin removal with the Proteus NoEndo Spin Column Kit (Protein Ark, USA). The endotoxin units (EU) determined by ToxinSensor Chromogenic LAL Endotoxin Assay Kit (GenScript, Piscataway, USA) were < 0.05 EU/mg protein.

#### Quantification of cellular Met phosphorylation *in situ*

AML12 immortalized mouse hepatocytes were obtained from ATCC (CRL-2254). AML12 cells were maintained in Dulbecco's Modified Eagle Medium (DMEM)/Ham's F-12 supplemented with 10% fetal bovine serum (FBS), Insulin-Transferrin-Selenium supplements (Sigma-Aldrich, St. Louis, USA), and 40 ng/ml dexamethasone (Sigma-Aldrich). AML12 cells were seeded at 10,000 cells per well in a 96-well black μClear-plate (Greiner Bio-One, Austria) and cultured in a culture medium for 24 h. Cells were stimulated with each protein in a culture medium for 10 min, washed with ice-cold PBS, fixed with 4% paraformaldehyde in PBS for 30 min, washed with PBS, and blocked with 5% goat serum, 0.02% Triton X-100 in PBS for 30 min. Met activation was detected using an anti-phospho-Met (Tyr1234/1235) antibody (1:1,000 dilution, D26, Cell Signaling Technologies) and a horseradish peroxidase (HRP)-conjugated anti-rabbit goat antibody (1:1,000 dilution, Dako/Agilent, USA), followed by measurement of chemiluminescence developed with ImmunoStar LD reagent (Fujifilm, Japan) using an ARVO MX plate reader (Perkin Elmer, USA).

#### Pull-down assay

The binding of peptide-grafted Fc variants to mouse Met_ECD_ was evaluated by pull-down assay. To this end, soluble ectodomain fragment of mouse Met (residues 1-931) C-terminally fused with PA-tag was expressed and captured onto the beads immobilized with anti-PA tag antibody NZ-1 (Fujifilm, Japan).^87^ After brief washing, the beads were further incubated with the culture supernatants containing peptide-grafted Fc variants. Bound proteins were then eluted by adding an SDS-containing buffer and analyzed by SDS-PAGE. The binding activity of each grafted Fc was assessed by its band intensity. Fc variants showing nonspecific binding similar to the control Fc protein were considered non-binders.

#### Flow cytometry

The binding of peptide-grafted Fc variants to human HEK293T (ATCC, USA) cells and AML12 cells was detected using flow cytometry. Cells were detached from dishes by a brief treatment with 0.025% trypsin and 1 mM EDTA, plated at 200,000 cells per well, and incubated with peptide-grafted Fc variants diluted at ∼10 μg/ml in 100 μl Expi expression medium on ice for 1.5 h. After washing twice with ice-cold PBS, cells were incubated with Alexa Fluor 488-labeled goat antihuman IgG (1:400 dilution, A11013, Thermo Fisher Scientific, USA) on ice for 30 min, then analyzed on an EC800 system (Sony, Tokyo, Japan). The gate on forward scatter vs. side scatter was set to include all cell populations and exclude debris and dead cells. The data were analyzed with FlowJo software (BD Biosciences, USA).

#### Western blotting and Phospho-RTK Array

AML12 cells were serum-starved in DMEM with 0.2% BSA for 3 h, stimulated with HGF (0.01∼1 nM), Fc(mML1)B3 (1∼100 nM), Fc (100 nM), or unstimulated (None) in DMEM with 0.2% BSA for 10 min, washed with ice-cold PBS. For western blotting, AML12 cells were lysed in 400 μl of lysis buffer (50 mM Tris, 150 mM NaCl, 1% Triton-X100, 1% NP-40, 10% glycerol, 1 mM phenylmethylsulfonyl fluoride, 1 mM Na_3_VO_4_, 1 mM NaF, 1 mM EDTA, and protease inhibitors). The lysates were passed through a 27G needle and 0.45 μm filter and centrifuged. Protein concentrations in lysates were measured by bicinchoninic acid assay (Thermo Fisher Scientific, USA). The supernatants were subjected to western blotting for phosphorylated Erk1/2 (T202/Y204) (1:1,000 dilution, D13.14.4E, Cell Signaling Technology, Danvers, USA), Erk1/2 (1:1,000 dilution, 137F5, Cell Signaling Technology), phosphorylated Akt (S473) (1:1,000 dilution, D9E, Cell Signaling Technology), Akt (1:1,000 dilution, 11E7, Cell Signaling Technology), GAPDH (1:1,000 dilution, 14C10, Cell Signaling Technology) or immunoprecipitation with an anti-Met antibody (5 μg for 400 μl lysates, B-2, Santa Cruz Biotechnology, Dallas, USA) and western blotting for Met (1:1,000 dilution, 25H2, Cell Signaling Technology) and phosphorylated Met (Y1234/Y1235) (1:1,000 dilution, D26, Cell Signaling Technology), and HRP-conjugated secondary antibody (Dako/Agilent, USA) followed by measurement of chemiluminescence developed with ImmunoStar LD reagent (Fujifilm, Japan) using Image Reader LAS-3000 mini Ver. 2.2 (Fujifilm, Tokyo, Japan) or a Fusion Solo S image acquisition system (Vilber Lourmat, Germany). For Phospho-RTK Array, AML12 cells were lysed in a lysis buffer (RayBiotech, USA) and passed through a 0.45 μm filter and centrifugation. The lysates (1 mg) were analyzed using the Mouse RTK phosphorylation Antibody Array C1 Kit (RayBiotech, USA).

#### Cell growth assay

AML12 cells were plated at 2,000 cells per well in 96-well plates in DMEM/Ham's F-12 medium supplemented with 2.5% FBS with or without hHGF or Fc(mML1)B3. The culture media with or without hHGF or Fc(mML1)B3 were replaced every third day. After 5 days, viable cells were measured by a 3-(4,5-dimethylthiazol-2-yl)-5-(3-carboxymethoxyphenyl)-2-(4-sulfophenyl)-2H-tetrazolium, inner salt (MTS) assay (Promega, USA).

#### Pharmacokinetics

Serum concentrations up to 14 days were determined in wild-type C57BL/6 female mice (9 weeks-old, 18 to 21 g, obtained from Japan SLC, Shizuoka, Japan) after a single subcutaneous (SC) injection of 5.0 mg/kg Fc(mML1)B3 (*n* = 4) diluted in PBS. To compare these serum concentrations against HGF, C57BL/6 female mice (9 weeks-old, 25 to 27 g) (*n* = 3) were injected intravenously (IV) with 2.0 mg/kg human recombinant HGF (Kringle Pharma, Osaka, Japan) diluted in PBS. Blood was drawn after 3 hours, 1, 3, 7, 10, and 14 days from the submandibular vein using a Goldenrod animal lancet (Bio Research Center) and processed into serum; allowed to clot at room temperature for 30 min and centrifuged at 1,200 g for 15 min; the supernatant was kept at -80°C until use. The serum concentrations of Fc(mML1)B3 were determined using a human immunoglobulin recognition immunoassay (human IgG ELISA quantitation set, Bethyl Laboratories, Montgomery, USA). The serum concentrations of HGF were determined using an in-house-developed ELISA.^74^ Briefly, a 96-well flat-bottom plate (Thermo Fisher Scientific, UK) was coated with 2 μg/mL of a rabbit polyclonal anti-human HGF antibody as the capture antibody in Carbonate-Bicarbonate Buffer pH 9.0 at 4°C overnight. After washing with TBST, a blocking agent consisting of 3% BSA in TBST was applied and allowed to incubate for 1 h at room temperature. Following blocking, both samples and the standard curve were incubated for 1 h at room temperature. Subsequently, the plate was washed again, and incubated with 2 μg/mL of biotinylated-rabbit anti-human HGF antibody, for 1 h at room temperature. After another washing step, the samples were incubated with streptavidin conjugated with HRP (1/5000 dilution) (Thermo Fisher Scientific, USA) at room temperature for 1 h. The plate was then washed, and TMB substrate (Cell Signaling, USA) was added to allow a reaction at 37°C. The reaction was stopped with 2M H_2_SO_4_, and the absorbance was finally measured at 450nm.

Additionally, C57BL/6 female mice (7 weeks-old, 15 to 20 g, obtained from Japan SLC, Shizuoka, Japan) were injected subcutaneously with 5.0 mg/kg of either Fc (*n* = 6) or Fc(mML1)B3 (*n* = 7) diluted in PBS. Then, three mice from each group were sacrificed after 24 h and the remaining mice after 120 h. Blood samples were collected from the inferior vena cava. The liver was carefully removed, weighed, and transferred into a tube with a bead and 1 mL of lysis buffer composed of 20 mM (pH 7.5) Tris, 150 mM NaCl, 100 mM NaF, 10 mM EDTA, 1 mM phenylmethylsulfonyl fluoride, 1 mM Na_3_VO_4_, 1 mg/mL Aprotinin, and 2% Triton-X100. Then the tubes with lysis buffer and liver pieces were loaded into a tissue homogenizer (Bertin Biotechnologies, France) following the manufacturer's recommendations. The resultant mixture was centrifuged at 4,000 rpm for 5 min and then 15,000 rpm at 4°C for 15 min; the supernatant was kept at -80°C until use. The blood samples were processed into serum, as mentioned above. The serum and lysate buffer concentrations of Fc and Fc(mML1)B3 were determined using a human immunoglobulin recognition immunoassay (human IgG ELISA quantitation set, Bethyl Laboratories, Montgomery, USA). To calculate the concentrations in the liver, ng/g liver was considered to be ng/ml.

#### *In* vivo Met activation

C57BL/6J inbred female mice (8 weeks-old, 17 to 22g) were obtained from Japan SLC. The mice were intravenously injected in the lateral tail vein with either Fc (4.55 mg/kg, *n* = 1), Fc(mML1)B3 (5 mg/kg, *n* = 1), recombinant human HGF (0.5 mg/kg, *n* = 1), or PBS (*n* = 1). After 10 min, the mice were euthanized and perfused with PBS. The liver was then removed, placed in 4% paraformaldehyde for 24 h, transferred into sucrose solutions of 10% (2 h), 20% (2 h), and 30% overnight at 4°C, and finally embedded in Tissue-Tek O.C.T embedding medium (Sakura Finetek Japan, Osaka, Japan) to create frozen blocks. The frozen tissue specimens were sectioned at 4 μm and mounted. To prepare the samples, 4% paraformaldehyde was applied for 30 min. After washing with ultrapure water and rinsing with PBS, the specimens were blocked with 5% BSA and 0.3% Triton X-100 in PBS for 1 h at room temperature. Subsequently, the specimens were incubated overnight at 4°C with a rabbit anti-phospho-Met antibody (1:200 dilution, Abcam, Cambridge, UK). After washing, the samples were incubated with the secondary antibody (Alexa Fluor 488-conjugated Goat anti-Rabbit; 1:200 dilution, Abcam, Cambridge, UK) for 1 h at room temperature, protected from light. Following another wash, the specimens were allowed to react with 4′,6-diamidino-2-phenylindole, (DAPI) (1:1000) for 30 min at room temperature in the dark before being mounted. Images were captured using a Keyence BZ-800 microscope, with five random captures at x20. The positive signal was measured in each image and averaged for quantification.

#### Mouse NASH model CDAHFD, HFD

C57BL/6J inbred male mice (7 weeks-old, 16 to 20 g) were purchased from Japan SLC and acclimated for one week. Then, they were given a CDAHF diet (L-amino acid diet with 60 kcal% fat, 0.1% methionine, and no added choline) for 12 weeks, 10 mice were sacrificed at this point to obtain data before treatment (Pretreat.) as reference. Then, a high-fat diet (HFD, 60 kcal% fat, and crystalline amino acids, Research Diets) was used instead of the CDAHFD. Mice were divided into two groups: control (*n* = 10) treated with subcutaneous injections of 4.3 mg/kg Fc, and the experimental group (*n* = 10) treated with 5 mg/kg of Fc(mML1)B3 once a week for 2 weeks. The body weight was recorded every week. Then, the animals were euthanized, blood samples were collected from the inferior vena cava, and the liver was carefully removed, weighed, and then collected for RNA, protein, and histological analysis.

#### Mouse NASH model CDAHFD, standard diet

Like the above-described model, C57BL/6J inbreed male mice (7 weeks-old, 16 to 20 g) were fed a CDAHF diet for 12 weeks. Three mice were sacrificed at this point to obtain data before treatment (Pretreat.). One day before starting the treatment, a standard diet was used instead of the CDAHFD. Mice were divided into two groups: control (*n* = 9) treated with subcutaneous injections of 4.3 mg/kg Fc, and the experimental group (*n* = 7) treated with 5 mg/kg of Fc(mML1)B3 at day 1, 4, and 7 (total doses = 3). Posterior tissue analysis was conducted in the same way as the NASH model CDAHFD, HFD. Mice were sacrificed after 10 days of treatment.

#### Histological analysis

Liver tissue was fixed overnight at 4°C in 4% paraformaldehyde. The next day, some tissue pieces were transferred into 70% ethanol overnight and then passed through increasing ethanol concentrations until paraffin embedding to make paraffin tissue blocks, which were cut using a microtome (Yamato, Japan) at 4 μm and put onto APS-coated slides for picrosirius red and immunohistochemistry analysis. The remaining pieces were transferred into 15%, 20%, and 30% sucrose overnight to finally make frozen blocks using a Tissue-Tek O.C.T embedding medium (Sakura Finetek Japan, Osaka, Japan). These frozen tissue specimens were sectioned at 4 μm in a Leica CM1950 Cryostat, (Leica Biosystems, Switzerland) and then put onto slides. 4% paraformaldehyde was applied to the samples for 30 min and washed using ultrapure water to be stained immediately with Oil Red O. The paraffin slides were allowed to dry overnight at 37°C. Then, when they were going to be used for staining, they were melted at 60°C for 10 min and then transferred to xylene, decreasing ethanol concentrations until water.

#### Picrosirius red

A stain kit (Polyscience, USA) was used according to the manufacturer's recommendations. Briefly, phosphomolybdic acid was applied on the samples of the deparaffinized slides for 2 min, then Picrosirius red stain for 60 min, and 0.1 N Hydrochloride Acid for 2 min. The slides were transferred into 70% ethanol, and then through ascending concentrations of ethanol and xylene, and finally covered. The images were analyzed using Image J software (NIH, Bethesda, USA). Every slide contained a piece of more than one hepatic lobe. A picture where the whole slide could be seen was taken and uploaded to Image J, the scale was set at 1 mm. Using the RGB Stack command, the image was changed into grayscale, and the green channel was used to set the threshold between 150-190. Each whole tissue piece from different lobes on the slide was measured to obtain the % positive area, which was averaged with all the slides from the analyzed groups.

#### Hematoxylin and Eosin

The deparaffinized slides were treated with Mayer’s Hematoxylin (Fujifilm, Japan) for 5 min, followed by rinsing under running water for 10 min. Subsequently, they were stained with Eosin (Fujifilm, Japan) for 2 min before being passed through ascending concentrations of ethanol and xylene. Finally, the slides were covered. Images of the tissues were captured using an Olympus BX51 microscope (Olympus, Japan).

#### Oil Red O

The frozen samples were quickly transferred into 60% isopropanol and covered with Oil Red O (Nacalai-Tesque, Kyoto, Japan) solution for 15 min, dipped in 60% isopropanol, washed in water, and finally covered with aqueous mounting medium gel. The obtained images were analyzed using Image J. Briefly, the 200 μm scale was set as a reference, the image was changed to 8-bit and adjusted to eliminate inter-droplet areas, the threshold was set at 190, and the positive percentage was calculated by including the whole area of the sections of different lobes and averaged with all the slides for the analyzed groups in duplicate.

#### Immunohistochemistry

For macrophage detection in liver tissue of paraffin slides, the slides were antigen-retrieved with Proteinase K (Takara, Kusatsu, Japan) treatment, endogenous peroxidase quenching by 3% hydrogen peroxide, blocked with 3% BSA/PBS, incubated with rat monoclonal F4/80 (2 μg/ml, A3-1, Bio-Rad, Hercules, USA) for overnight, incubated with an anti-rat IgG HRP-conjugated antibody (1:200, R&D systems), detected with 3,3'-diaminobenzidine (Vector Laboratories, Newark, USA), and counterstained with Mayer's Hematoxylin (Fujifilm, Japan) for 3 min. Similarly, α-SMA-expressing cells were detected using an anti-α-SMA antibody (1:1,000, Abcam, Cambridge, UK), the antigen retrieval was performed using pH 8.0 TE Buffer (10 mM Tris, 1 mM EDTA), and Histofine® Simple Stain MAX PO (Nichirei, Tokyo, Japan) was used to detect the first antibody. To observe proliferating cells, we used anti-Ki-67 antibody (1:200 dilution, SP6, Abcam, Cambridge, UK). pH 6.0 Citrate Buffer was used for the antigen retrieval step, and an anti-rabbit IgG HRP-conjugated antibody (1:200, Dako/Agilent, USA) was used as the second antibody. Pictures from the tissues were taken using an Olympus BX51 microscope (Olympus, Japan). Image analysis from the stained slides was done using Image J, the Immunohistochemistry Image Analysis Toolbox. The image was converted into 8-bit, and a threshold was set to quantify positive areas from 7 (10x) fields/liver for α-SMA and 20 fields (10x) liver.

#### TUNEL assays

To detect apoptotic cells and observe their morphology, the TUNEL Assay Kit-HRP-DAB (Abcam, Cambridge, UK) was used following the manufacturer’s protocol for paraffin-embedded tissue sections. After rehydration, the specimens were permeabilized using Proteinase K (1:100) for 20 min at room temperature, followed by a quenching step in 3% hydrogen peroxide for 15 min. The samples were then covered with equilibration buffer for 30 min, and the labeling reaction was performed with TdT Enzyme diluted at 1:40 for 1.5 h at 37°C. After terminating the reaction, a blocking step was performed for 10 min. Detection was carried out by applying the provided conjugate for 30 min, followed by DAB solution for 15 min at room temperature. Counterstaining was performed using Methyl Green from the same kit. The slides were then transferred to xylene and covered. The images were analyzed using Image J software (NIH, Bethesda, USA). Non-overlapping images were captured at 40x magnification for each sample, with seven images used per group. The number of TUNEL-positive cells and the total number of cells were counted in each image, differentiating hepatocytes from non-hepatocytes based on morphology. The quantifications were performed by dividing the number of TUNEL-positive hepatocytes by the total number of hepatocytes, TUNEL-positive non-hepatocytes by the total number of non-hepatocytes, and TUNEL-positive cells by the total number of cells in each image and expressing these values as percentages. Additionally, to detect apoptotic cells, the CF596-TUNEL Assay kit (CUSABIO, Houston, USA) was used following the protocol provided by the manufacturer for fresh frozen tissue sections. The tissue sections were washed and fixed with 4% paraformaldehyde for 30 min, followed by permeabilization with PBS containing 0.2% Triton X-100. The TUNEL reaction was carried out overnight at 4°C, and the sections were stained with DAPI (1:1000) for 30 min. Images at x20 magnification were captured using a Keyence BZ-800 microscope (KEYENCE CORPORATION, US), with 10 pictures taken per mouse's liver. Quantification involved dividing the number of apoptotic bodies by the DAPI-positive cells in each field, and an average was calculated across the 10 fields.

#### N-Terminal Procollagen III propeptide

To detect mouse PIIICNP (N-Terminal Procollagen III Propeptide), a kit was used (CUSABIO, Houston, USA), following the manufacturer's protocol. Briefly, the required coated wells were removed from the provided 96-well plate, then the standards and samples were applied and left at 37°C for 2 h, followed by incubation with the biotin-antibody at 37°C for 1 h, and then avidin-HRP. Finally, TMB was applied and stopped before reading at 450 nm.

#### ELISA assays

To measure inflammation related markers the following kits were used; Mouse TNF-α ELISA MAX Standard kit (Biolegend, USA), Mouse RANTES/CCL5 ELISA kit (Thermo Fisher Scientific, USA), TGF-β1 Human/Mouse Uncoated ELISA kit (Thermo Fisher Scientific, USA), and IL-6 Uncoated ELISA kit (Thermo Fisher Scientific, USA) following the manufacturers' protocols.

#### Triglycerides measurement and ALT

To measure the triglyceride content in the liver, 100−300 mg of liver pieces were incubated overnight at 55°C in ethanolic KOH, treated with 1M MgCl_2_, and the resultant supernatant was used to measure the triglycerides through a LabAssay Triglyceride Kit (GPO-DAOS method, Fujifilm) following the manufacturer's protocol. To measure the ALT in serum, an Alanine Aminotransferase Activity Assay Kit (Sigma-Aldrich, USA) following the manufacturer's recommendations was performed.

#### RNA sequencing and data analysis

Total RNA from RNA*later* (Thermo Fisher Scientific, USA)-treated livers from mice was prepared using QIAzol lysis reagent (QIAGEN, Germany) and sent to Bioengineering Lab Co., Ltd. (Sagamihara, Japan) for RNA quality control and RNA sequencing on a DNBSEQ-G400 sequencer (MGI Tech, Shenzhen, China). cDNA libraries were prepared using the MGIEasy RNA Directional Library Prep Set (MGI Tech) and evaluated using the AATI Fragment Analyzer (Advanced Analytical Technologies, Ankeny, USA). The cDNA libraries were circularized using MGIEasy Circularization Kit (MGI Tech), prepared as DNA nanoballs by DNBSEQ-G400RS High-throughput Sequencing Kit (MGI Tech), and followed by massively parallel sequencing (2×100 bp) on a DNBSEQ-G400 sequencer. The RNA-sequence reads were processed based on the reference genome mm10. Adaptor sequence trimming and quality check were done using Fastp, and removal of ribosomal RNA was performed using Bowtie2 (ver 2.4.5). Reads were aligned to the reference genome using the STAR tool (ver 2.7.10a). The aligned reads were then counted using featureCounts in the Subread package (ver. 2.0.3). Then, the integrated web application for Differential Expression and Pathway analysis of RNA-Seq data, iDEP 0.951^80^ was used to perform the Differentially Expressed Gene (DEGs) analysis with the DESeq2 package. Significant values are written in the legends of the respective figures. The protein-protein interaction networks of DEGs were determined by discriminating upregulated and downregulated genes and were visualized on the String online database (https://string-db.org/; version 11.5). The resulting networks were exported to Cytoscape (version 3.9.1)^83^ using the plug-in StringApp (version 2.0.1).^84^

#### Quantitative Reverse transcription (RT)-PCR

Total RNA from RNA*later* (Thermo Fisher Scientific, USA)-treated livers from mice was prepared using QIAzol lysis reagent (QIAGEN, Germany). cDNA was synthesized with a SuperScript™ III Reverse Transcriptase (Thermo Fisher Scientific, USA). *CXCL1*, *CCL2*, *CCL5*, *TGF-b,* and *actin b* (*ACTB*) mRNA were detected by quantitative RT-PCR using the ViiA™ 7 Real-Time PCR System (Thermo Fisher Scientific, USA). The primer pairs and probes were obtained from the TaqMan assay reagents library (Thermo Fisher Scientific, USA).

#### Safety analysis

C57BL/6J inbred female mice (8 week-old, 17 – 22 g) were subcutaneously administered either Fc (4.55 mg/kg, *n* = 3), Fc(mML1)B3 (5 mg/kg, *n* = 3), or PBS (*n* = 3) once a week for two consecutive weeks to observe the effects of the HGF mimetic. After the two-week treatment period, the mice were euthanized, and the liver, spleen, kidneys, and lungs were carefully harvested and transferred to 4% paraformaldehyde overnight at 4°C. Subsequently, the tissues underwent a series of ethanol washes with increasing concentrations, ultimately being embedded in paraffin. Sections, 4 μm thick, were cut from these tissues and utilized for hematoxylin and eosin staining.

### Quantification and statistical analysis

Prism 6.0d (GraphPad Software) was used for graphing and statistical analysis. Relevant statistical methods for individual experiments are detailed within the figure legends. Differences between groups were analyzed by Student's t-test, and the level of significance was *p* < 0.05. Data are presented as the mean ± SEM or SD. ns = not significant. ∗ *p* < 0.05, ∗∗ *p* < 0.01, ∗∗∗ *p* < 0.001.
