# Supplementary material for: A cyclic peptide-grafted Fc with hepatocyte growth factor functionality ameliorates hepatic fibrosis in a non-alcoholic steatohepatitis mouse model
Source: iScience. 2024 Jul 2;27(8):110426. doi: 10.1016/j.isci.2024.110426 (PMC11300919; doi:10.1016/j.isci.2024.110426)
Supplement: Document S1. Figures S1–S8 [file mmc1.pdf]

iScience, Volume *27*

Supplemental information

A cyclic peptide-grafted Fc with hepatocyte growth factor functionality ameliorates hepatic fibrosis in a non-alcoholic steatohepatitis mouse model

Nichole Marcela Rojas-Chaverra, Ryu Imamura, Hiroki Sato, Toby Passioura, Emiko Mihara, Tatsunori Nishimura, Junichi Takagi, Hiroaki Suga, Kunio Matsumoto, and Katsuya Sakai

Supplemental figures and legends

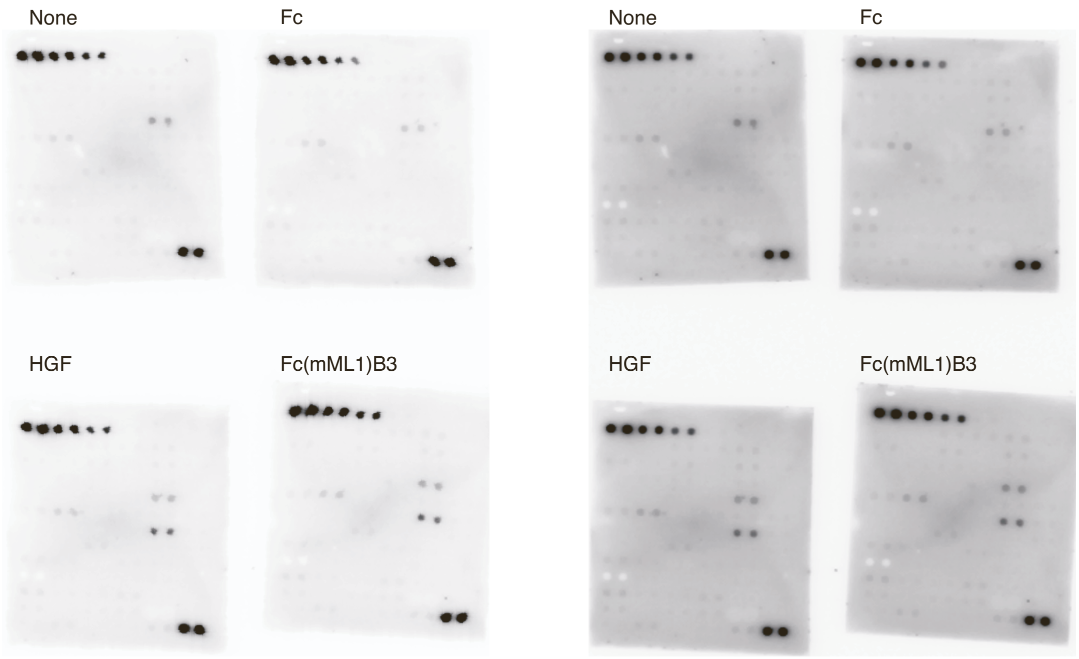

	A	B	C	D	E	F	G	H	I	J	K	L
1	POS1	POS1	POS2	POS2	POS3	POS3	ABL1	ABL1	ACK1	ACK1	ALK	ALK
2	NEG	NEG	NEG	NEG	Axl	Axl	Blk	Blk	BMX	BMX	Btk	Btk
3	Csk	Csk	Dtk	Dtk	EGFR	EGFR	EphA1	EphA1	EphA2	EphA2	EphA3	EphA3
4	EphA4	EphA4	EphA5	EphA5	EphA6	EphA6	EphA7	EphA7	EphA8	EphA8	EphB1	EphB1
5	EphB2	EphB2	EphB3	EphB3	EphB4	EphB4	EphB6	EphB6	ErbB2	ErbB2	ErbB3	ErbB3
6	ErbB4	ErbB4	FAK	FAK	FER	FER	FGFR1	FGFR1	FGFR2	FGFR2	FGFR2 (α isoform)	FGFR2 (α isoform)
7	Fgr	Fgr	FRK	FRK	Fyn	Fyn	Hck	Hck	HGFR	HGFR	IGF-IR	IGF-IR
8	Insulin R	Insulin R	Itk	Itk	JAK1	JAK1	JAK2	JAK2	JAK3	JAK3	LCK	LCK
9	LTK	LTK	Lyn	Lyn	MATK	MATK	M-CSFR	M-CSFR	MUSK	MUSK	NGFR	NGFR
10	PDGFR-α	PDGFR-α	PDGFR-ß	PDGFR-ß	PYK2	PYK2	RET	RET	ROR1	ROR1	ROR2	ROR2
11	ROS	ROS	RYK	RYK	SCFR	SCFR	SRMS	SRMS	SYK	SYK	Tec	Tec
12	Tie-1	Tie-1	Tie-2	Tie-2	TNK1	TNK1	TRKB	TRKB	TXK	TXK	NEG	NEG
13	Tyk2	Tyk2	TYRO10	TYRO10	VEGFR2	VEGFR2	VEGFR3	VEGFR3	ZAP70	ZAP70	POS4	POS4

POS = Positive Control Spot
NEG = Negative Control Spot
BLANK = Blank Spot

Figure S1. Uncropped images of Western blots, related to Figure 4A.

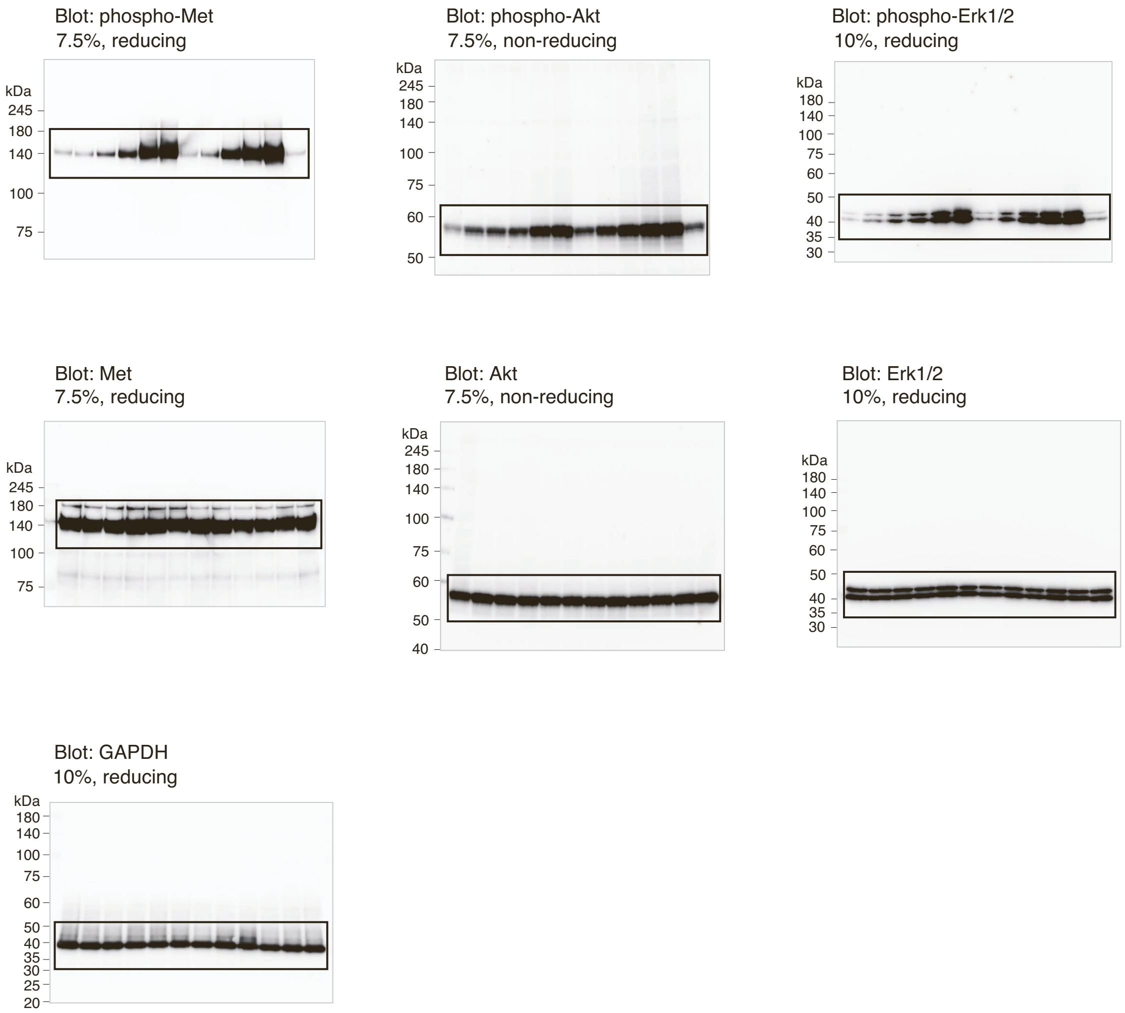

Figure S2. Uncropped images of Western blots, related to Figure 4B.

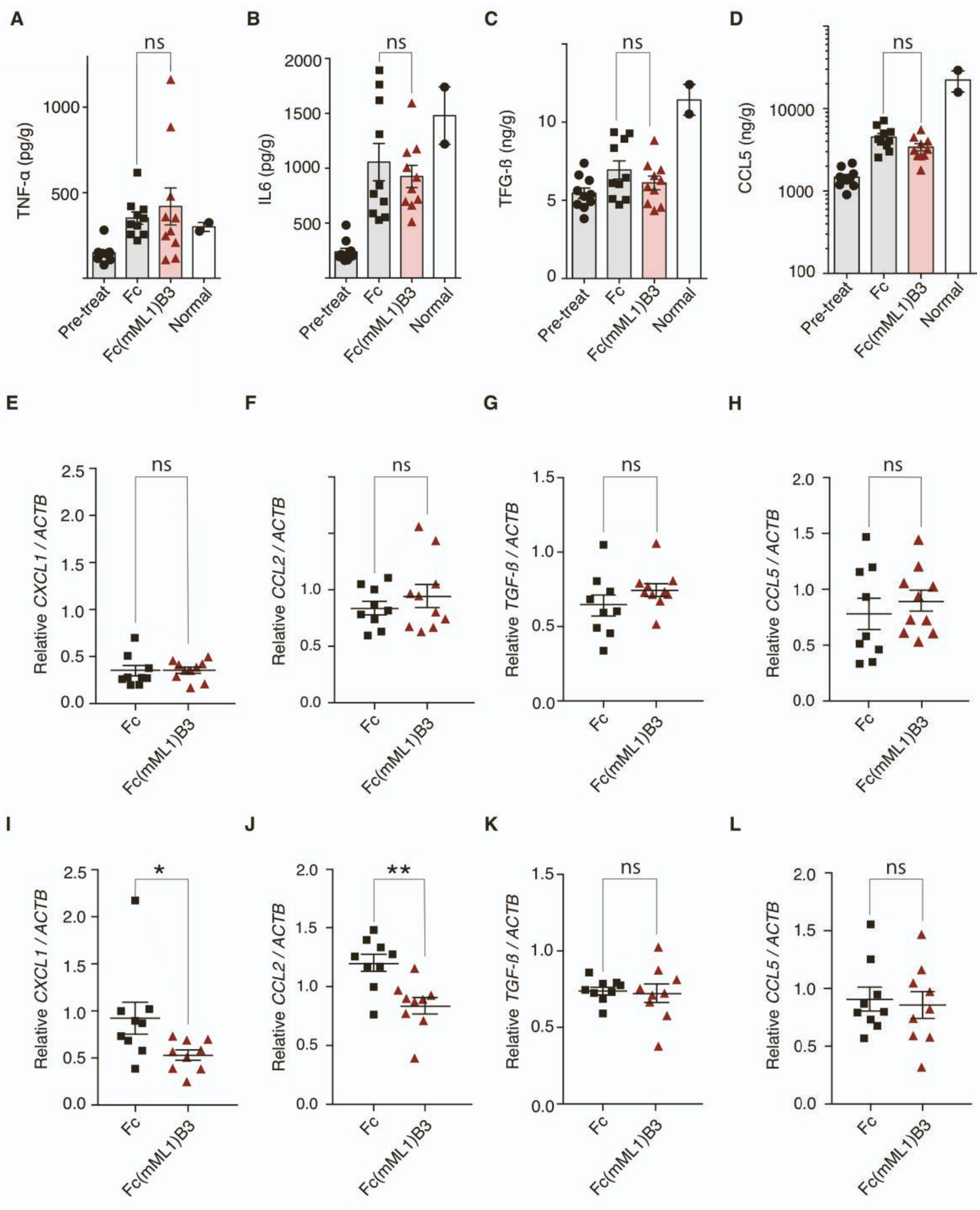

Figure S3. Inflammatory markers, related to Figure 6. C57BL/6 mice fed CHAHFD for 12 weeks (Pretreatment group, $n = 10$), and 2 additional weeks of HFD with 5 mg/kg of Fc(mML1)B3 ($n = 10$) or 4.3 mg/kg of Fc ($n = 10$) once a week. Quantification in liver lysate of (**A**) TNF-α, (**B**) IL-6, (**C**) TGF- β and (**D**) CCL5. qRT-PCR quantification comparing Fc and Fc(mML1)B3 given HFD of: (**E**) *CXCL1*, (**F**) *CCL2*, (**G**) *TGF- β* and (**H**) *CCL5*. qRT-PCR quantification comparing Fc and Fc(mML1)B3 given Standard diet of: (**I**) *CXCL1*, (**J**) *CCL2*, (**K**) *TGF-β* and (**L**) *CCL-5*. Data are presented as the mean ± SEM. ns = not significant. * $p < 0.05$, ** $p < 0.01$.

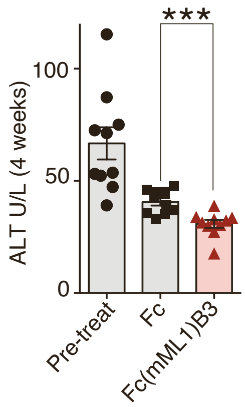

Figure S4. Serum ALT levels after 4 weeks of treatment, related to Figure 6. Serum ALT levels were quantified after 4 weeks of treatment with either Fc (*n* = 10) or Fc(mML1)B3 (*n* = 10) once a week while they were being fed the HFD. Data are presented as the mean ± SEM, *** $p < 0.001$.

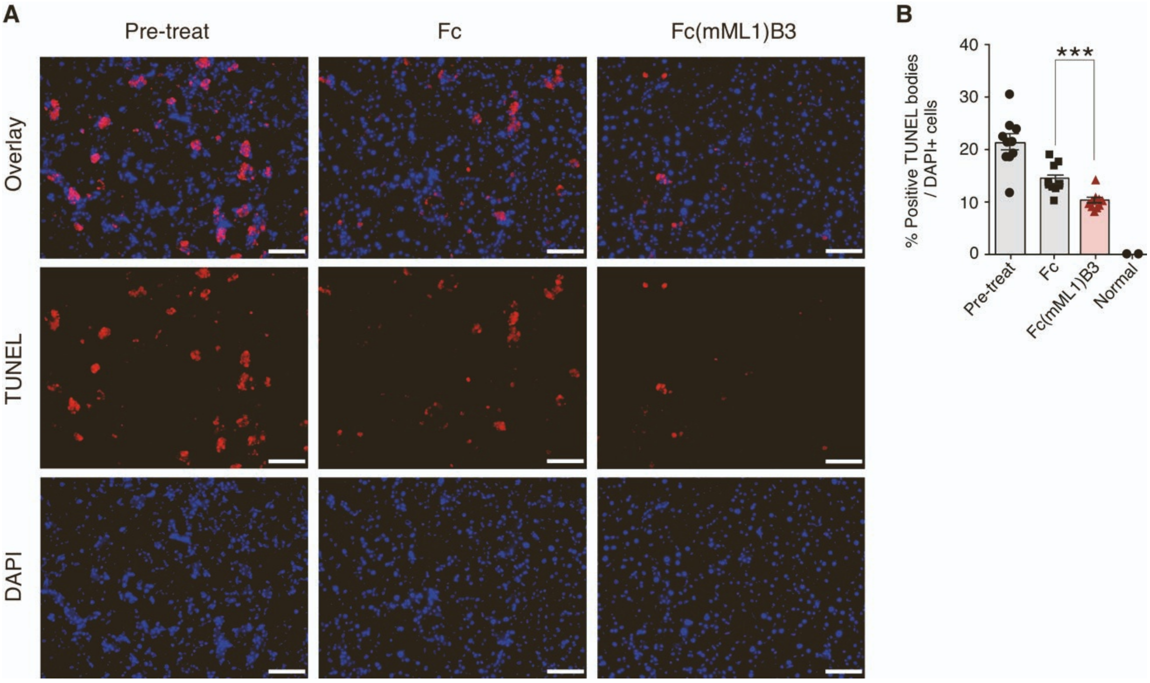

Figure S5. Reduction of apoptosis in the liver by Fc(mML1)B3 in the murine NASH model, related to Figure 6. (**A**) Representative images of TUNEL assay. Scale bars: 100 μm. (**B**) Quantitative analysis of apoptotic bodies (red) / DAPI + cells (blue). Data are presented as the mean ± SEM. *** $p < 0.001$.

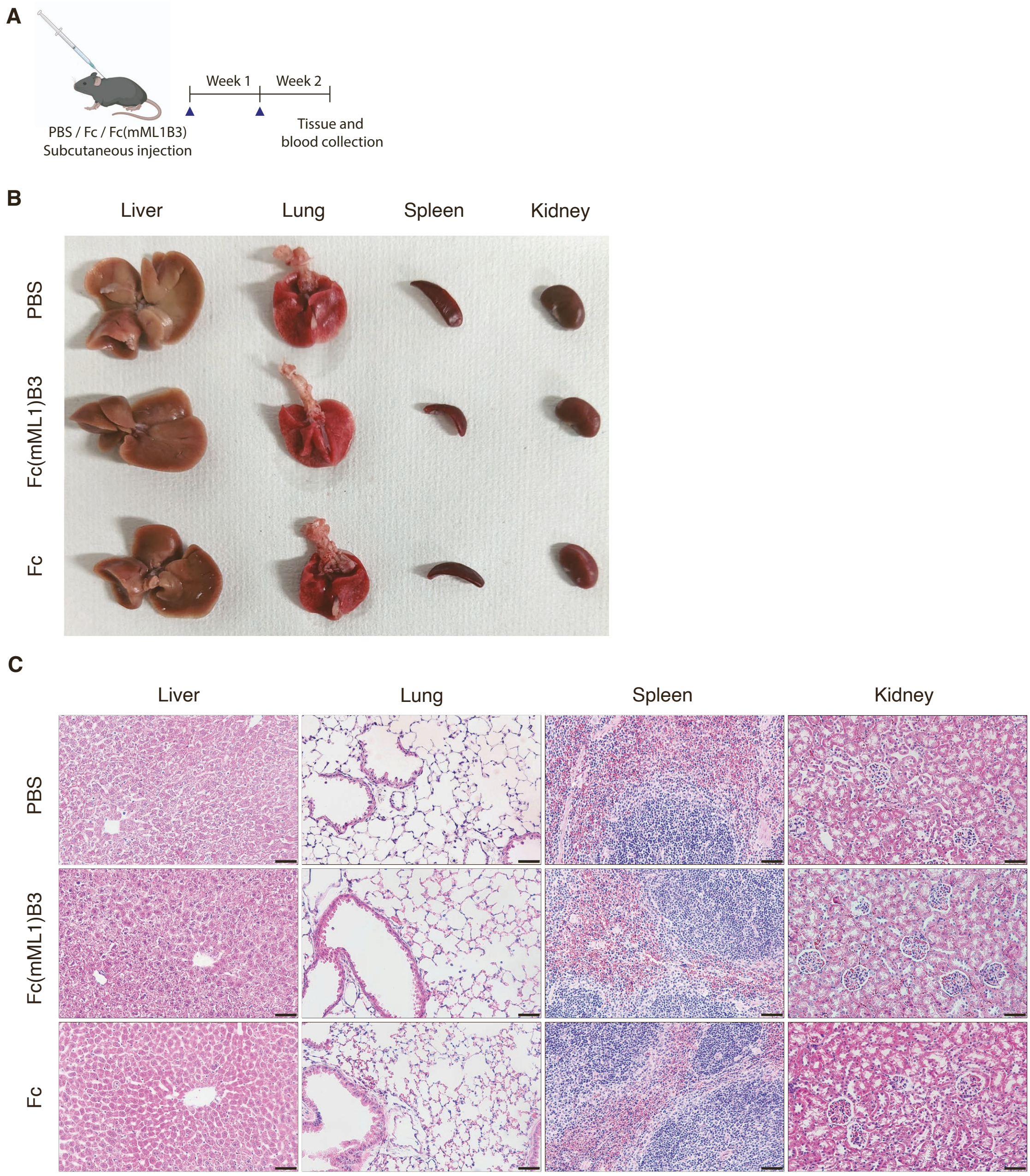

Figure S6. Safety analysis, related to Figure 6. (**A**) C57BL/6J mice were subcutaneously administered either Fc (4.55 mg/kg), Fc(mML1)B3 (5 mg/kg), or PBS once a week for two consecutive weeks (*n* = 3 per group). **(B)** Gross anatomy of the harvested organs. **(C)** Representative images of tissues stained with Hematoxylin and Eosin. Scale bars: 50 μm.

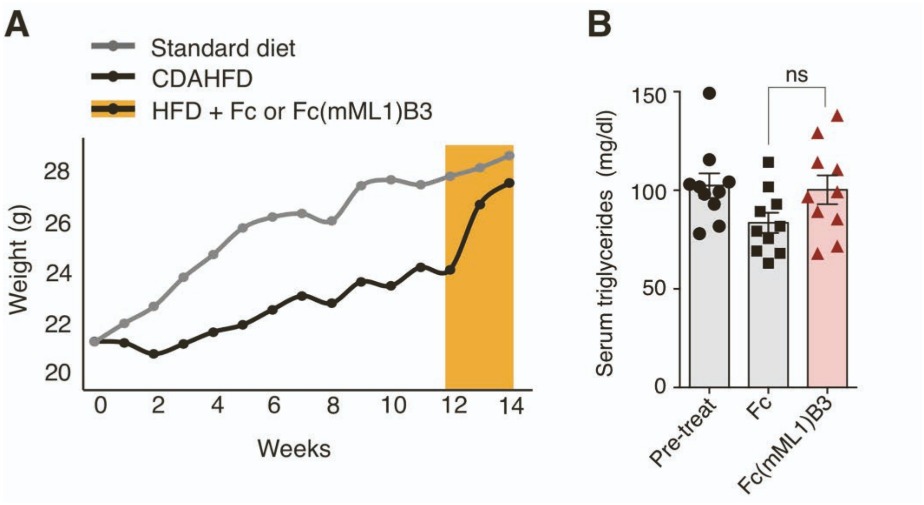

Figure S7. Body weight change of male C57BL/6 mice under HFD condition, related to Figure 6. (**A**) C57BL/6 mice fed CHAHFD for 12 weeks (black line, $n = 30$). The increase in weight over the 2-week experimental period is shown as a black line with an orange shadow in mice fed HFD with subcutaneous administration of either Fc ($n = 10$) or Fc(mML1)B3 ($n = 10$). There was no difference in weight gain between the two regimes (HFD + Fc vs. HFD + Fc(mML1)B3, $p = 0.2$). (**B**) Triglyceride concentrations in serum after 2 weeks of HFD + Fc or Fc(mML1)B3. The serum triglyceride levels were slightly higher in the Fc(mML1)B3 group but the difference was not significant ($p = 0.07$). The serum triglyceride levels were not higher than those measured in mice of the same age fed the standard diet (170 mg/dL, $n = 2$). Data are presented as the mean ± SEM. ns = not significant.

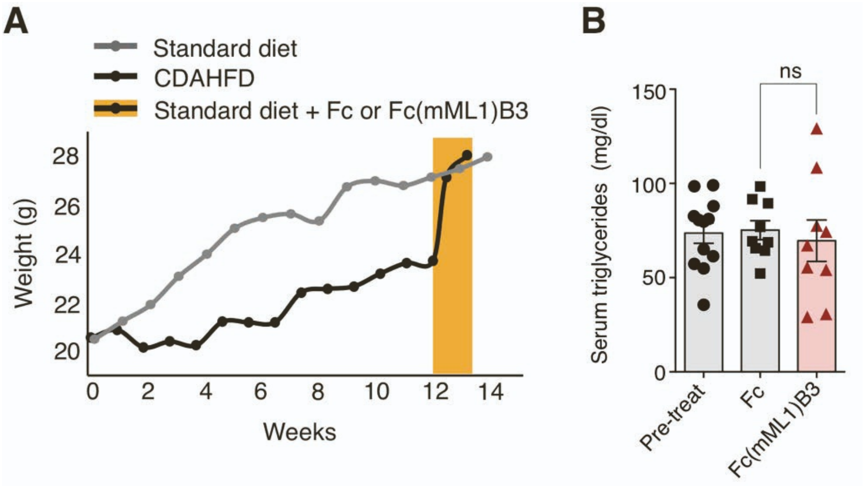

Figure S8. Body weight change of male C57BL/6 mice under standard diet condition, related to Figure 7. (A) C57BL/6 mice were fed CDAHFD for 12 weeks (black line, *n* = 16). The black line with an orange shadow shows the increase in weight over 10 days of mice fed a standard diet with subcutaneous administration of either Fc (*n* = 9) or Fc(mML1)B3 (*n* = 7). Mice in both groups had similar body weights to the group fed the standard diet during the same period (27.9 – 29.9 g, *n* = 2). Average body weights were 27.5 g in the Standard diet + Fc group and 28.5 g in the Standard diet + Fc(mML1)B3 group (*p* = 0.006) after 10 days of treatment. (**B**) Serum triglyceride levels were not different between the two groups (*p* = 0.6). Data are presented as the mean ± SEM. ns = not significant.
